# Exploring the Remarkable Diversity of Culturable *Escherichia coli* Phages in the Danish Wastewater Environment

**DOI:** 10.3390/v12090986

**Published:** 2020-09-04

**Authors:** Nikoline S. Olsen, Laura Forero-Junco, Witold Kot, Lars H. Hansen

**Affiliations:** 1 Department of Environmental Science, Aarhus University, Frederiksborgvej 399, 4000 Roskilde, Denmark; sno@plen.ku.dk; 2 Department of Plant and Environmental Sciences, University of Copenhagen, Thorvaldsensvej 40, 1871 Frederiksberg C, Denmark; laura.junco@plen.ku.dk

**Keywords:** bacteriophage, wastewater, *Escherichia coli*, diversity, genomics, taxonomy, coliphage

## Abstract

Phages drive bacterial diversity, profoundly influencing microbial communities, from microbiomes to the drivers of global biogeochemical cycling. Aiming to broaden our understanding of *Escherichia coli* (MG1655, K-12) phages, we screened 188 Danish wastewater samples and isolated 136 phages. Ninety-two of these have genomic sequences with less than 95% similarity to known phages, while most map to existing genera several represent novel lineages. The isolated phages are highly diverse, estimated to represent roughly one-third of the true diversity of culturable virulent dsDNA *Escherichia* phages in Danish wastewater, yet almost half (40%) are not represented in metagenomic databases, emphasising the importance of isolating phages to uncover diversity. Seven viral families, *Myoviridae*, *Siphoviridae*, *Podoviridae, Drexlerviridae, Chaseviridae, Autographviridae,* and *Microviridae,* are represented in the dataset. Their genomes vary drastically in length from 5.3 kb to 170.8 kb, with a guanine and cytosine (GC) content ranging from 35.3% to 60.0%. Hence, even for a model host bacterium, substantial diversity remains to be uncovered. These results expand and underline the range of coliphage diversity and demonstrate how far we are from fully disclosing phage diversity and ecology.

## 1. Introduction

Phages are important ecological contributors, renewing organic matter supplies in nutrient cycles and driving bacterial diversity by enabling co-existence of competing bacteria by “Killing the winner” and by serving as genomic reservoirs and transport units [1,2]. Phage genomes are known to contain auxiliary metabolism genes (AMGs), toxins, and virulence factors [3,4,5,6,7]. Through lysogeny and transduction, they can transfer metabolic traits including antibiotic resistance to their hosts and can confer immunity against homologous phages [1].

Still, despite their ecological role, potential as antimicrobials and the fact that they carry a multitude of unknown genes with great potential for biotechnological applications, phages are vastly understudied. Around 10,000 phage genomes have now been published [8]. Though the number increases rapidly, we may have merely scratched the surface of the expected diversity. It is estimated that at least one billion bacterial species exist [9]. Hence, only phages targeting a tiny fraction of potential hosts have been reported. Efforts to disclose the range and diversity of phages targeting a single host have revealed a stunning display of diversity. The most scrutinized phage host is the *Mycobacterium smegmatis*, for which the Science Education Alliance Phage Hunters program has isolated more than 4700 phages and fully sequenced 680. These represent 30 distinct phage clusters [10,11]. This endeavour has provided a unique insight into viral and host diversity, evolution, and genetics [12,13,14,15]. No other phage host has been equally targeted, but numerous *Escherichia coli* phages (coliphages) have been isolated. The International Committee on Taxonomy of Viruses (ICTV) currently recognises 276 phage species originally isolated from *Escherichia* species [16], while 733 *Escherichia* phage assemblies are listed in the European Nucleotide Archive (ENA) [17]. 

As phages are expected to have evolutionary potential to migrate across microbial populations, host species may not be an ideal indicator of relatedness, but it serves as an excellent starting point to explore phage diversity. The hierarchical classification of phages is complicated by the high degree of horizontal gene transfer. Consequently, several classification systems have been proposed [18,19,20]. We may not yet have reached a point where it is reasonable to establish the criteria for a universal system [20]. Nonetheless, a system enabling a mutual understanding and exchange of knowledge is needed. Accordingly, we have classified our phages as per the ICTV guidelines [21].

Here, we aim to expand our understanding of coliphage diversity, by screening for coliphages targeting a single strain of *E. coli*. Earlier studies on coliphage diversity have explored other aspects. Grose & Casjens (2014) did a comprehensive *in silico* study on 337 genomes of tailed phages infecting *Enterobacteriaceae* to characterise the known diversity [8]. Jurczak-Kurek et al. (2016) isolated 60 coliphages from sewage samples on a single *E. coli* strain and thoroughly assessed the physiological diversity but did not sequence any of the coliphages [22]. Korf et al. (2019) used 29 individual *E. coli* strains from various sources to isolate, characterise, and sequence 50 diverse tailed coliphages including representatives of novel phage lineages, verifying that there is still something to discover [23]. Mathieu et. al., (2020) explored the presence of virulent and temperate coliphages in 648 faecal samples from 1-year-old children, revealing interesting compositional trends likely to impact gut microbiota dynamics [24].

In this study we applied the High Throughput Screening (HiTS) screening method on 188 wastewater samples using the K-12 MG1655 strain as host. This approach favours virulent, culturable dsDNA phages with a large burst size and a short latency period [25]. We hypothesised that using a non-pathogenic lab-strain like MG1655 would provide a broad diversity of phages as opposed to wildtype strains which are likely better equipped for avoiding a wide array of successful phage infections. Hence, we expected the screening to yield coliphages that were easy to work with but also distinct enough to expand the number of known coliphages.

## 2. Materials and Methods 

The screening for coliphages was performed with the HiTS method as described in [25], though instead of direct plaque sequencing [26], lysates of wells giving rise to plaques were sequenced. In short, an overnight enrichment (37 °C) was performed in microplates with *E. coli*, media, and wastewater (0.5 mL/well); the next day, the enrichments were filtrated (0.45 µm), re-inoculated (∼1 μL), and re-incubated overnight (37 °C); on the third day, a second filtration (0.45 µm) and a spot-test (soft-agar overlay) were performed to indicate positive wells.

### 2.1. Sample Bacteria and Media

Inlet wastewater samples (188) were collected (40–50 mL) at two- to four time-points during July and August 2017 from 48 Danish wastewater treatment plants (WWTPs) geographically distributed in Denmark. The samples were centrifuged (9000× *g*, 4 °C, 10 min) and the supernatant filtered (0.45 μm) before storage in aliquots (−20 °C) until screening. The host bacterium was *E. coli* (MG1655, K-12), and the medium, Lysogeny Broth (LB), was amended with CaCl_2_ and MgCl_2_ (final concentration 10 mM).

### 2.2. Sequencing and Genome Characterisation

DNA extractions, clean-up (ZR-96 Clean and Concentrator kit, Zymo Research, Irvine, CA, USA), and sequencing libraries (Nextera^®^ XT DNA kit, Illumina, San Diego, CA, USA) were performed according to manufacturer’s protocols with minor modifications as described in Kot et al. (2014) [26]. The libraries were sequenced as paired-end reads on an Illumina NextSeq platform with the Mid Output Kit v2 (300 cycles). The obtained reads were trimmed and assembled in CLC Genomics Workbench version 10.1.1. (CLC BIO, Aarhus, Denmark). Overlapping reads were merged with the following settings: mismatch cost: 2, minimum score: 15, gap cost: 3 and maximum unaligned end mismatches: 0, and then assembled de novo. Additional assemblies were constructed using SPAdes version 3.12.0 [27]. Gene prediction and annotation were performed using a customized RASTtk version 2.0 [28] workflow with GeneMark [29], with manual curation and verification using BLASTP [30], HHpred [31], and Pfam version 32.0 [32], or *de novo* annotation was performed using VIGA version 0.11.0 [33] based on DIAMOND searches (RefSeq Viral protein database) and HMMer searches (pVOG HMM database). All genomes were assessed for antibiotic resistance genes, bacterial virulence genes, restriction-modification genes, and auxiliary metabolism genes (AMGs) using ResFinder 3.1 [34,35], VirulenceFinder 2.0 [36], Restriction-ModificationFinder 1.1 (REBASE) [37], and VIBRANT version 1.0.1 [38], respectively. 

### 2.3. Bioinformatics

Nucleotide (NT) and amino acid (AA) similarities were calculated using tools recommended by the ICTV [21], i.e., BLAST [30] for identification of the closest relative (BLASTn when possible, discontinuous megaBLAST (word size 16) for larger genomes), and Gegenees version 2.2.1 [39] for assessing phylogenetic NT (BLASTn) and AA (tBLASTx) distances of multiple genomes, with fragment size 200 bp and step size 100 bp. Intergenomic nucleotide sequence similarity and aligned genome fractions between all isolated phage species were plotted with VIRIDIC [40]. NT similarity was determined as percentage query cover multiplied by percentage identity. Novel phages were categorised according to ICTV taxonomy. The criterion of 95% DNA sequence similarity for demarcation of species was applied to identify novel species representatives and to determine uniqueness within the dataset. Evolutionary analyses for phylogenomic trees were conducted in MEGA7 version 2.1 (default settings) [41]. These were based on the large terminase subunit (*Caudovirales*), a gene commonly applied for phylogenetic analysis [42,43] and on the DNA replication gene (*gpA*) (*Microviridae*). The NT sequences were aligned by MUSCLE [44] and the evolutionary history inferred by the Maximum Likelihood method based on the Tamura-Nei model [45]. The trees with the highest log-likelihood and are shown. Pairwise whole genome comparisons were performed with Easyfig 2.2.2 [46] (BLASTn), curated by adding color-codes and identifiers in Inkscape version 0.92.2. The R package iNEXT [47,48] in R studio version 1.1.456 [49] was used for rarefaction, species diversity (q = 0, datatype: incidence_raw), extrapolation thereof (estimadeD), and estimation of sample coverage. The visualisation of genome sizes and GC contents was prepared in Excel version 16.31. Blast+ 2.9.0 [50] was used to perform a NT search of the coliphages (queries) against a database with the IMG/VR v2.0 database sequences [51] and the human gut virome database (GVD) v 1.7 [52]. Reads from metagenomes and metaviromes were mapped using bbmap 38.22 [53]. Genome breadth and depth coverage was calculated using genomecov from BEDtools 2.28.0 [54] and BamM 1.7.3 [55], respectively.

## 3. Results and Discussion

### 3.1. Wastewater Coliphages Are Remarkably Diverse

The sequenced coliphages were analysed strictly *in silico,* focusing on their relatedness to known phages, taxonomy, and distinctive characteristics. The genome assemblies had a coverage of ×20-12122 with an average of ×390.5 (Table S1). The genome screening algorithms identified no homologs of known virulence or antibiotic resistance genes. Though not a definitive exclusion, this is interpreted as a reduced risk of presence, a preferable trait for phage therapy. The majority of genes identified when screening for AMGs code for phage DNA modification pathways (Table S2). 

**Figure 1:**
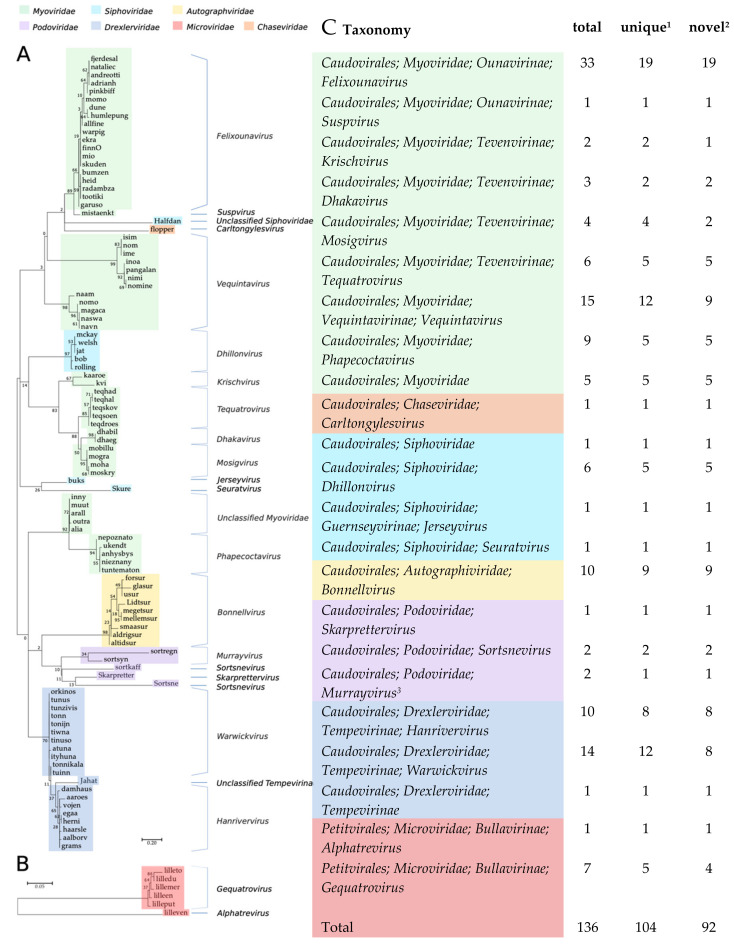
Phylogenetic trees, bootstrap: 100, scalebar: substitutions per site. (**A**) *Caudovirales* based on large terminase subunit *terL* (Maximum log Likelihood: −1801.27). (**B**) *Microviridae* based on the DNA replication protein gene *gpA* (Maximum log Likelihood: −3922.55). (**C**) Taxonomic distribution of phages identified in 94 Danish wastewater samples, based on similarity to closest related and the ICTV Master Species list. ^1^ ≤95% similarity to other phages in the dataset. ^2^ ≤95% similarity to other phages in the dataset and the NCBI GenBank. ^3^ The *Murrayvirus* genus has by mistake been classified as *Siphoviridae*, but a proposed move to *Podoviridae* will be included in the 2021 ICTV ratification.

The isolation method (HiTS) favours easily culturable plaque-forming virulent phages [25]. Still, even though we screened wastewater samples, which is a commonly used source for isolation of coliphages, we identified 136 coliphages of which 92 differed by ≥5% from published phage genomes and some with nucleotide (NT) similarities as low as 29% (Table 1). Based on Blastn analyses and the 95% nucleotide similarity demarcation, 104 of the coliphages are unique phage species (Table 1, Figure 1). Based on DNA homology and phylogeny, the 104 unique coliphages group into 14 distinct clusters and 7 single phages (Figure 1 and Figure S1). Coliphages were identified in samples from 44 of the 48 WWTPs (Table S1). There was no substantial difference in phage diversity distribution between samples of urban or rural origin (Figure S2). Samples without coliphages likely reflect the crude nature of the screening method and in some cases sequence or assembly issues and not actual absence. From the majority of positive samples (*n* = 58) a single phage was sequenced, though some lysates held more than one phage (28 lysates: 2 phages, 6 lysates: 3 phages, 1 lysate: 4 phages, Table S1).

**Figure 2:**
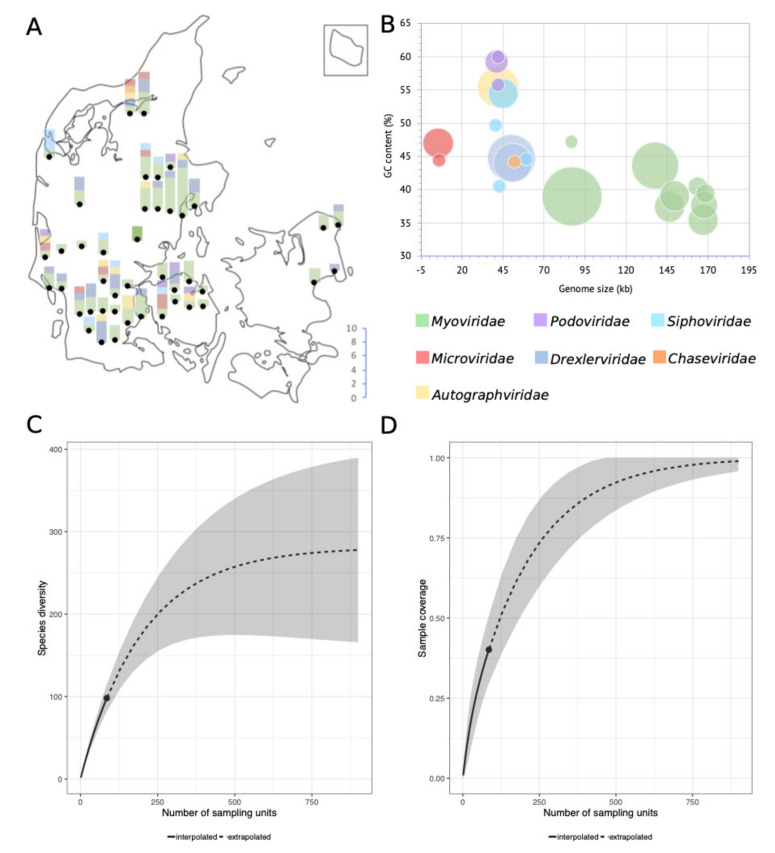
(**A**) Number of phages and taxonomic family distribution per WWTP, as well as approximate geographical location thereof. (**B**) Bubble-diagram of the 104 unique coliphage species displaying genome size and GC content distribution; the area of the bubbles indicates the number of phages. (**C**) Sample completeness curve with confidence intervals (0.95). (**D**) Sample-size-based rarefaction and extrapolation curve with confidence intervals (0.95). Sampling units in figures C and D denote isolated phages (*n* = 136).

The 95% nucleotide identity demarcation of species is an arbitrary delimitation. It does not consider the biological importance of the non-identical sequence parts and imposes a discrepancy between the demarcation of species depending on genome size. However, it provides a means to quantify and compare relatedness enabling estimations of, e.g., culturable virulent coliphage species richness in the Danish wastewater environment (Figure 2. An extrapolation of species richness (q = 0) predicts a total of 311 distinct species (requiring a sample size of ∼900 phages) (Table S3). The relatively small sample size in this study (*n* = 136) may subject the estimation to a large prediction bias. The sampling-method also introduces bias by selecting for abundance, latency, and burst size, thereby potentially underestimating diversity. Sequencing and assembly methods as well as the choice of a host further reduce the number of detected phage genomes. Nonetheless, the results indicate the minimal diversity of culturable virulent dsDNA coliphages (MG1655, K-12) in Danish wastewater, estimated to be as a minimum in the range of 183 to 350 unique phage species (Figure 2, Table S3). 

The diversity of tailed dsDNA coliphages is well documented [8,22,23] and it is to be expected that a screening of nearly 200 wastewater samples would yield hitherto unknown phages. However, considering the use of only a single host strain and a crude isolation method ensuring that only a single or the few most successful phage(s) from each sample were sequenced, then the degree of novelty and diversity revealed is remarkable and verifies our hypothesis, as well as the efficiency of the HiTS method for exploring diverse phages of a single host [25].

### 3.2. Taxonomy of the 104 Novel Coliphages

Based on the confirmed morphology of closely related phages, six different families of the order *Caudovirales* are represented; *Myoviridae* (57.4%), *Drexlerviridae* former *Siphoviridae* (18.4%), *Autographviridae* former *Podoviridae* (7.4%), *Siphoviridae* (6.6%)*, Podoviridae* (3.7%) and *Chaseviridae* (0.7%), as well as *Microviridae* (5.9%), order *Petitvirales* (Figure 1). A similar distribution of coliphages from surface water, manure, sewage, and animal faeces was found by Korf et al. (2019); 70% *Myoviridae,* 22% *Siphoviridae,* and 8% *Podoviridae* [23]. Grose & Casjens (2014) also identified more clusters belonging to the *Myoviridae,* than the *Siphoviridae* and the fewest for the *Podoviridae* when analysing genomes of *Caudovirales* infecting *Enterobacteriaceae* [8]. Jurczak-Kurek et al. (2016) found more *Siphoviridae* than *Myoviridae*, but also found the *Podoviridae* to be the least abundant coliphages in sewage [22]. However, these distributions likely reflect abundance distributions of culturable phages and not necessarily natural abundances.

#### 3.2.1. Fifty-Five *Myoviridae* Species

The 55 *Myoviridae* phage species represent the greatest span in genome sizes, from the *Suspvirus* mistaenkt (86.7 kb) to the *Dhakavirus* dhaeg (170.8 kb) (Figure 2B), and all, except the *Krischvirus*, code for tRNAs (Table 1). The *Myoviridae* group into eight distinct clusters and one single phage (mistaenkt), representing three subfamilies; *Tevenvirinae*, *Vequintavirinae* and *Ounavirinae*, in addition to the *Phapecoctaviruses* and a cluster of six unclassified *Myoviridae* (Figure 1, Table 1). The *Tevenvirinae* represent four genera, two krischviruses, five tequatrovirus, two dhakaviruses and two mosigviruses notable for their ability to perform arabinosylation of hmC [56]. 

**Figure 3:**
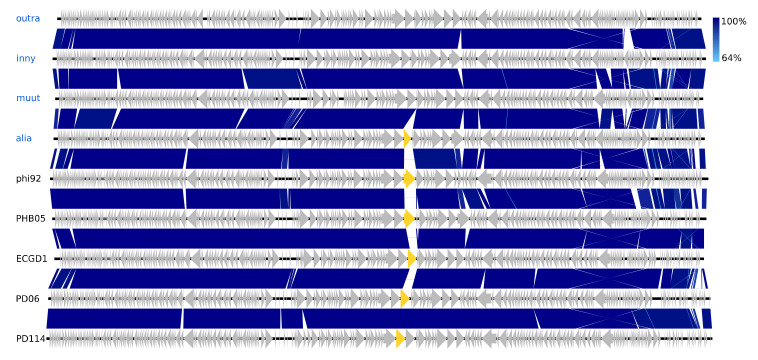
Pairwise alignment of the unclassified *Myoviridae* phage species from this study (blue text) and their closest relatives (black text), the color bars between genomes indicate percent pairwise similarity (Easyfig, BlASTn). The genes marked with yellow code for a tailspike (gene 143 in phi92).

The isolated *Vequintavirinae* are all vequintaviruses closely related (91.1–93.8%, BLAST) to classified species and were identified in samples from 12 of the 48 WWTPs. All of the *Ounavirinae* but the Suspvirus mistaenkt are felixounaviruses (89.7–93.9%, BLAST). The *Felixounavirus* is a relatively large genus with 16 recognized species isolated from *Escherichia* and *Salmonella*. In this study, felixounaviruses were identified 33 times in samples from no less than 23 WWTPs, indicating that they are ubiquitous in the Danish wastewater environment and that they are easily cultivated, though the method prevents assessment of relative preponderance. Felixounaviruses often have broad within-genus host ranges, and isolates have been shown to be able to rapidly expand their host range when challenged, co-coinciding with mutations in the long tail gene [57,58]. Five of the *Myoviridae* are members of the newly announced genus *Phapecoctavirus* with substantial similarity (86–90%, BLAST) to the type species Escherichia phage phAPEC8 (JX561091) [23,59]. The five phages in the last of the *Myoviridae* clusters are an even more homogeneous group than the phapecoctaviruses (Figure 3 and Figure S1). All five are closely related (92–95%, BLAST) to the same five unclassified *Enterobacteriaceae* phages vB_Ecom_PHB05 (MF805809), vB_vPM_PD06 (MH816848), ECGD1 (KU522583), phi92 (NC_023693), and vB_vPM_PD114 (MH675927) [60,61]; this group of nine phages is distinct (<44% NT similarity, BLAST) from all other described phages and thus represents a yet to be classified genus, presumably with the first sequenced phage phi92 as type species. Phi92 was isolated in 1982 and has been thoroughly characterised; it has a broad across-genus (*Salmonella, Escherichia*) host range enabled by multiple divergent tail fibres and can infect both non-capsulated and encapsulated hosts as it has a unique endosialidase tailspike encoded by gene 143 [60,61]. Interestingly, this gene appears to be unique for phi92, though other versions of a putative tailspike are present at the same position in the genomes of alia, PHB05, ECGD1, and the two PD06 and PD114 phages (Figure 3). Both the phapecoctaviruses and the unclassified *Myoviridae* genomes code for a complete dTDP-rhamnose biosynthesis pathway. The presence of a dTDP-rhamnose biosynthesis pathway in the DNA metabolism region of phage genomes is peculiar; one possible explanation is that these phages utilize rhamnose for glycosylation of hydroxy-methylated NTs in the same manner as the T4-generated glucosyl-hmC [56].

#### 3.2.2. A New Addition to the Small Family *Chaseviridae*

**Figure 4:**
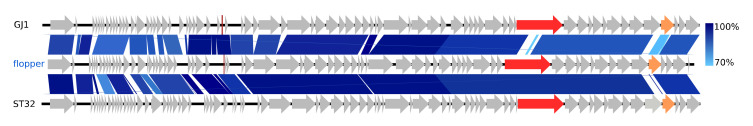
Pairwise alignment of the new *Carltongylesvirus* phage flopper (blue) and the *Carltongylesvirus* phages GJ1 and ST32; the colour bars between genomes indicate percent pairwise similarity (Easyfig, BlASTn). Genomes have been modified to have similar starting points. Genes coding for the putative tail tape measure protein (red) and a tail fiber (orange) are colorised.

The distinctive phage flopper only shares NT similarity (38.5–87%, BLAST) with ten other phages; it belongs to the newly established *Carltongylesvirus* (80.8–87% NT similarity, BLAST) of the new family *Chaseviridae.* This family currently has only nine species and the *Carltongylesvirus* only two species, Escherichia phage phiEcoM_GJ1 (EF460875) and Escherichia phage ST32 (MF044458). Both type species GJ1 and ST32 have broad within-genus host ranges [62,63]. NT similarity between flopper and GJ1 is partially low in the gene for the putative tail tape measure and also low between all three phage genomes in a tail fiber gene (Figure 4). The carltongylesviruses are unique in having characteristic *Myoviridae* morphology, i.e., icosahedral head, neck, and a contractile tail with tail fibres and also code for RNA polymerases, a feature otherwise characteristic to the T7-like phages of the *Autographiviridae* family [62,64,65].

#### 3.2.3. Six *Microviridae* of Two Genera

The single phage Lilleven and the five gequatroviruses belong to the subfamily *Bullavirinae,* family *Microviridae*, order *Petitvirales,* characterised by ssDNA non-enveloped icosahedral phages (Table 1). Lilleven is a novel species of the genus *Alphatrevirus,* closely related to (93.9% NT similarity, BLAST, 89–90% AA similarity, Gegenees) the *Alphatrevirus* Enterobacteria phage St1 (NC_012868) (Figure S3). The five gequatroviruses only differ from one another by single NT polymorphisms and in non-coding regions (Figure S3). They cluster and share genomic organisation and extensive NT similarity (92.6–94.5%, BLAST) with the unclassified *Microviridae* Escherichia phage SECphi17 (LT960607), but only have 59.1–67.9% NT similarity (BLAST) with recognised *Gequatrovirus* species, with which they have almost no sequence similarity in the region coding for the major spike protein (*gpG*), a distinctive marker of the subfamily *Bullavirinae* involved in host attachment (Figure S3, Table 1) [66]. However, considering the pronounced gene synteny between their relatively small genomes and a conserved AA similarity (62–64%, Gegenees), they are considered gequatroviruses.

The sequencing of the *Microviridae* is peculiar, as library preparation with the Nextera^®^ XT DNA kit applies transposons targeting dsDNA. However, during *Microviridae* infection, the host polymerase converts the viral ssDNA into an intermediate state of covalently closed dsDNA, which is then replicated in a rolling circle by viral replication proteins transcribed by the host RNA polymerase [67]. This intermediate state may have enabled the library preparation. The presence of host DNA (2.8–39.1% of reads) in the sequence results of these samples indicates an insufficient initial DNase I treatment (Table S4), which can be attributed to chemical inhibition or inactivation of the enzyme by adhesion to the sides of wells. Hence, it is reasonable to assume that the extracted microvirus DNA was captured as free dsDNA inside host cells during ongoing infections.

#### 3.2.4. Twenty *Drexlerviridae* Phages Including a New Linage Representative

**Figure 5:**
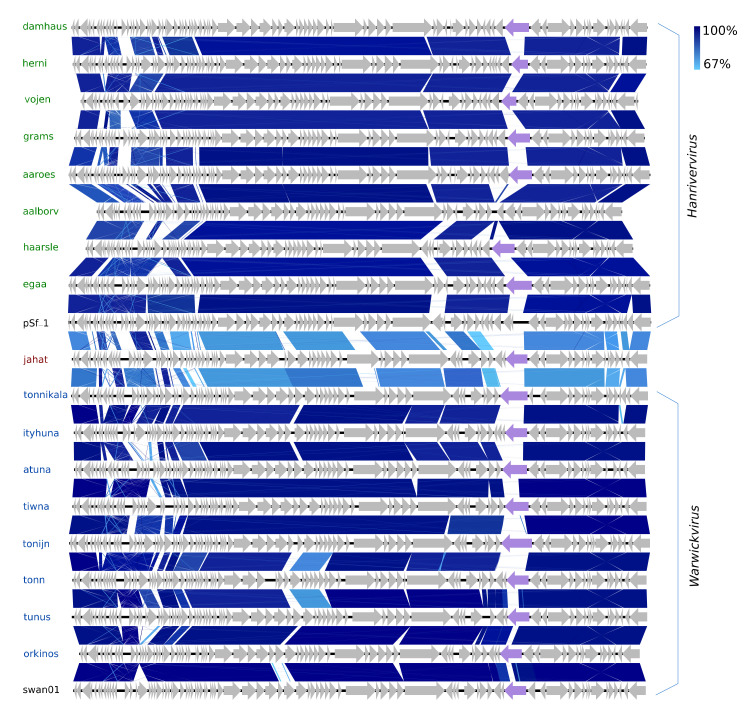
Pairwise alignment of the new *Hanrivervirus* phages (green text) and type species pSf-1 (black text), the new *Warwickvirus* phages (blue text), and type species swan01 (black text) and Jahat (brown text); the color bars between genomes indicate percent pairwise similarity (Easyfig, BlASTn). Genomes have been modified to have similar starting points. Coloured genes (purple) code for a putative tail fiber.

The 20 species of the new family *Drexlerviridae* represent a considerable expansion of the new subfamily *Tempevirinae* [68]. Eight of the *Drexlerviridae* belong to the new genus *Warwickvirus* (five species) with Escherichia virus swan01 as type species (LT841308), as they have ≥84.9% NT similarity (BLASTS) to recognised species thereof. The other eight are of the genus *Hanrivervirus* (NT: 86–90%, BLAST and AA: 77–85% Gegenees, Figure S4)*,* currently consisting of only the type species Shigella virus pSf-1 (NC_021331) isolated from the Han River in Korea [69]. The warwickviruses and hanriverviruses isolated in this study all have comparable genome sizes, GC contents, and gene organisation with the respective type species (Figure 5, Table 1). During their differentiation, many deletions and insertions of small hypothetical genes have occurred; most notable is a unique version of a putative tail-spike protein in seven of the new *Hanrivervirus* species and all of the new *Warwickvirus* species, indicating a variety of divergent host ranges (Figure 5). All the hanriverviruses code for (putative) dam, and Psf-1 is resistant against at least six restriction endonucleases [69], suggesting that these phages employ DNA methylation as a defence strategy. 

The last *Drexlerviridae* is Jahat. The warwickviruses and hanriverviruses form a monophyletic clade together with Jahat (Figure 1 and Figure S1). Even though Jahat has its own branch, this phage has gene synteny, slightly higher but comparable GC content, and shares an equal degree of NT similarity ≤68.7% with phages of both the *Hanrivervirus* and *Warwickvirus* (Figure 5). Hence, Jahat cannot with confidence be assigned to either genus but falls in between, barely different enough to represent its own genus—an indicator of the genetic continuum of phages challenging taxonomic delimitations.

#### 3.2.5. Eight *Siphoviridae* Species and a Novel Genus Representative

**Figure 6:**
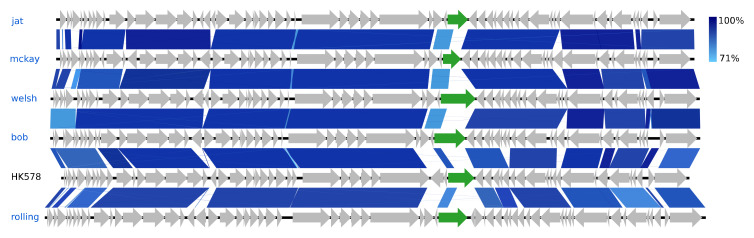
Pairwise alignment of the new *Dhillonvirus* phage species (blue text) and the type species HK578 (black text); the colour bars between genomes indicate percent pairwise similarity (Easyfig, BlASTn). Genomes have been modified to have similar starting points. Highlighted genes (green) code for a putative tail fiber.

The eight *Siphoviridae* species vary greatly in GC content, ranging from 44.6% (Skure) to 54.6% (welsh), but are quite similar in genome sizes, 49.7–54.6 kb (Figure 2). Five of these phages are of the genus *Dhillonvirus* as they have substantial NT similarity (77–80%, BLAST) and pronounced gene synteny with the type species Escherichia virus HK578. As with the hanriverviruses and warwickviruses, their genomes only differ in minor hypothetical genes and have limited NT similarity in a gene of highly variable length coding for a tail fiber (gp26 in HK578) (Figure 6), a phenomenon also observed in the dhillonviruses isolated by Korf et al. (2019), which correspondingly had divergent host ranges [23]. Each of the three remaining *Siphoviridae* represents a different genus. Based on NT similarity and the presence of the canonical 7-deazaguanine operon, Skure is of the 13-species genus *Seuratvirus*, while buks is assigned to the two-species genus *Jerseyvirus*, subfamily *Guernseyvirinae*.

**Figure 7:**
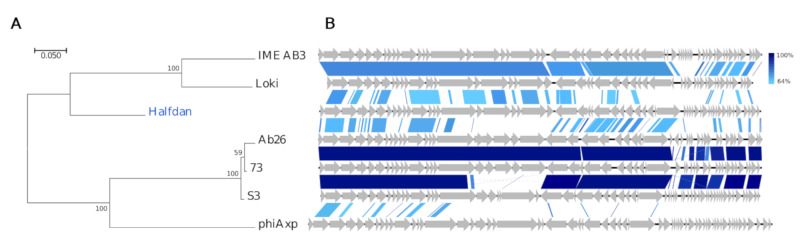
Comparisons of the new phage lineage representative Halfdan (blue text) and closest relatives (black text). (**A**) Phylogenetic tree, Maximum log Likelihood: −7678.71, bootstrap 100, large terminase subunit *TerL*, scalebar: substitutions per site. (**B**) Pairwise alignment of phage genomes; color bars between genomes indicate percent pairwise similarity (Easyfig, BlASTn). Genomes have been modified to have similar starting points.

Interestingly, the *Siphoviridae* Halfdan has only minuscule similarity with described phages (12–29%, BLAST). These entail two *Pseudomonas* phages vB_PaeS_SCUT-S3 (MK165657) and Ab26 (HG962376) [70], both *Septimatreviruses*, two *Acinetobacter* phages of the *Lokivirus* IMEAB3 (KF811200) and type species Acinetobacter virus Loki [71], and to a lesser degree the unclassified *Achromobacter* phage phiAxp-1 (KP313532) [72]. They have a common gene organization, yet their intra-Gegenees scores are low (≤1% BLASTn, <43% BLASTx, Figure S5), and NT similarity is negligent in roughly one-third of Halfdan’s 57 CDSs (Figure 7). The *TerL*-based phylogeny and AA similarity also indicate a distant relation, although grouping Halfdan closer with the lokiviruses (40–43%, Gegenees BLATSx) than the septimatreviruses (33–34%, Gegenees BLATSx) (Figure 7, Figure S5). Clearly Halfdan is distinct from all other described phages and hence the first phage sequenced of a new *Siphoviridae* genus. 

#### 3.2.6. Nine *Autographiviridae* Species

The nine *Autographiviridae* all have the hallmarks of this new family, i.e., unidirectionally encoded genes and RNA polymerases [65,68,73]. They belong to the genus *Bonnellvirus,* as they have conserved gene organisation, a similar GC content, and also share considerable NT similarity (69–93%, BLAST) with the type species Enterobacteria phage J8-65 (NC_025445) (Figure S6). The genomes of the nine new bonnellviruses and J8-65 are highly similar with differences primarily in small hypothetical genes, though Lidtsur codes for a unique version of tailspike colanidase (Figure S6). Lidtsur was deposited to the NCBI GenBank before the others and is currently the only one which is an ICTV-approved species representative. 

#### 3.2.7. Four *Podoviridae* Species Including Two Novel Genus Representatives

**Figure 8:**
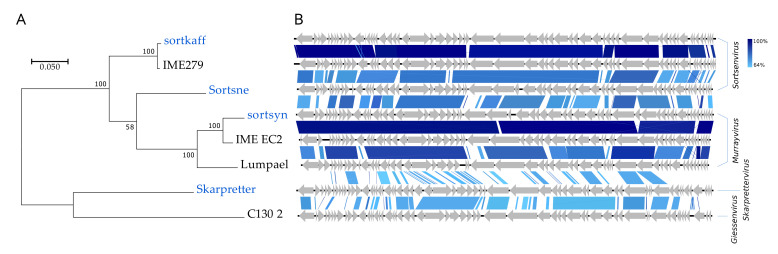
Comparisons of the *Podoviridae* phage species from this study (blue text) with closest relatives (black text). (**A**) Phylogenetic tree, Maximum log Likelihood: −8023.43, bootstrap 100, large terminase subunit *TerL*, scalebar: substitutions per site. (**B**) Pairwise alignment of phage genomes; color bars between genomes indicate percent pairwise similarity (Easyfig, BlASTn). Genomes have been modified to have similar starting points.

The four *Podoviridae* all have high (>59%) GC contents and represent no less than three distinct genera (Figure 2, Figure 8). Skarpretter is the type and only species of the genus *Skarprettervirus* [74]. Skarpretter is distinct from all described phages sharing only 38% NT similarity (BLAST) with the *Giessenvirus* Escherichia phage C130_2 (MH363708) isolated from cheese [75] (Figure 8 and Figure S7). Sortsne is the type species of the genus *Sortsnevirus* [74], currently consisting of only Sortsne and Klebsiella phage vB_KpnS_IME279 (MF614100); however, based on high NT similarity and conserved gene organization with IME279 (89.8%, BLAST), we suggest that sortkaff also belongs in *Sortsnevirus* (Figure 8 and Figure S7). The last *Podoviridae* sortsyn is of the new 2-species genus *Murrayvius* [76], as it shares a high degree of NT similarity and has conserved gene organization with the type species Enterobacteria phage IME_EC2 (KF591601) isolated from hospital sewage [77] (Figure 8).

### 3.3. The Wastewater Coliphages Are Largely Absent in Metaviromes

**Figure 9:**
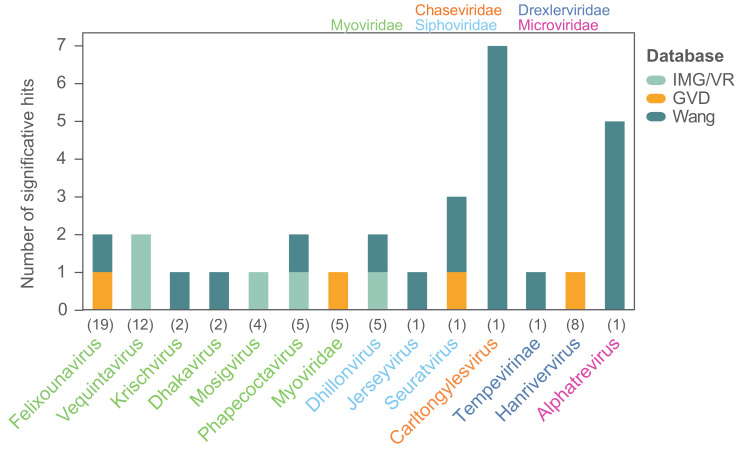
Significant hits for finding close relatives of the phages isolated in this study in three databases. Hits are defined as hits ≥80% genome coverage when blasting the coliphage genomes against the IMG/VR and GVD databases, and as mapped reads covering ≥70% of individual coliphage genomes when mapping reads from the Wang study. The coliphages are grouped according to genera, and numbers in parentheses denote the number of coliphage species representing each genus; only genera with significant hits are shown. Color-codes of genera denote taxonomic family.

In order to investigate the prevalence of the 104 coliphage species in different environments we mapped the reads of 510 metagenomes from studies of primarily Danish wastewater, pig, and human gut samples (Table S5) [78,79]. The threshold for significant hits was set as mapped reads covering ≥70% of a coliphage genome, and the distribution of the mapped reads was assessed to verify that this threshold ensured identification of closely related phages (Figure S8). No hits were found for any of the coliphages. This is likely a consequence of sequencing depth and sample preparation, as prior to sequencing, these metagenome samples were concentrated by centrifugation as a pellet or by CsCl gradient and the supernatant was either discarded or stored for future studies, and as a result, a large proportion of potential phage reads was omitted. Subsequently, we also searched for the coliphages in hundreds of metavirome datasets (Table S5) from Irish and Chinese faecal, human, animal, and water samples using the same read mapping method (Table S6). There were no hits to the human faecal contigs from Ireland [80], while 22 of the 104 coliphage genomes (21%) representing ten genera were covered by >70% by reads from 10 (mammals and birds) of the 38 (26%) Chinese Wang study libraries (Figure 9) [80]. For most phage genera, only reads from a single sample matched, though reads from five metaviromes (pet dog, pig, yak, and flamingo faeces) matched (>70% read coverage) with the *Alphatrevirus* Lilleven, and reads from seven metaviromes (dog, red panda, giant panda, non-human primate, masked civet, pig, and chicken faeces) matched (>70% read coverage) with the *Carltongylesvirus* flopper (Table S6). Finally, the genome sequences of the 104 coliphages (queries) were blasted against a database of 735,106 uncultured viral genomes (UVIGs) from the Integrated Microbial Genomes/Virus (IMG/VR) database, derived from a wide range of sample types including marine, freshwater, terrestrial, and hosts [51], as well as 13,203 UVIGs from human gut samples retrieved from the human gut virome (GVD) database (Table S7) [52]. The coliphage genomes were also blasted against the 8392 isolated virus genomes (iVGs) of the IMG/VR database and based on the observed alignment coverage distribution (Figure S9), significant matches were defined as those covering >80% of coliphage genomes. With this threshold, there were significant matches for 23 of the 104 (22%) coliphage genomes to four of the 735,106 (0.0005%) IMG/VR UVIG sequences (Figure 9, Table S7).

Only phages from 14 of the 24 taxonomic groups of coliphages from this study had matches in the virome databases assessed. For only 14 of the 62 coliphages with matches, a closely related phage could be identified in more than one virome. Although the coliphages are omnipresent and culturable in Danish wastewater, they are for a large part not represented in metagenomic data, and therefore these coliphage genomes provide valuable information. A lack of representation in metagenomic data could be caused by low natural abundance as this would result in insufficient sequencing depth for genome assembly within metagenomes/-viromes. The *Siphoviridae* Halfdan, the *Myoviridae* mistaenkt, and the phages of the *Drexlerviridae* genus *Warwickvirus,* the *Autographviridae* genus *Bonnellvirus,* all the *Podoviridae* genera, *Sortsnevirus, Murrayvirus* and *Skarprettervirus,* as well as the *Microviridae* genus *Gequatrovirus* did not match any UVIG sequences, nor did the reads from any virome cover ≥70% of their genomes. Surprisingly, the gequatroviruses were not detected in any of the gut-viromes, even though that apart from temperate and crAss-like phages, *Microviridae* dominates in human, mammal, and bird gut-microbiomes and that 860 *Microviridae* genomes were assembled from the assorted Wang et al. (2019) metaviromes [80,81]. However, due to the relatively small size of the *Microviridae* genomes, substitution of a single gene is enough to warrant a ∼5–33% difference in NT similarity, putting them below the set threshold for identification. The fact that both Halfdan and the bonnellviruses and all the *Podoviridae* represent novel genera with very few close relatives suggests that these lineages are under-sampled and not sufficiently abundant in the environments explored by metagenomic sequencing and assessed in this study. These findings underline the importance of isolating and sequencing individual phages in order to uncover diversity. It is plausible that phages selected for by plating techniques are not those that are naturally abundant; however, this cannot be concluded based on these results. Future studies should compare the diversity obtained by isolating to metagenomic sequencing of metaviromes of identical samples in order to establish the degree of discrepancy between these two methods.

## 4. Conclusions

By screening 188 wastewater samples, we identified 104 coliphages species (MG1655–K12), enabling us to predict the species richness of culturable virulent dsDNA coliphages in Danish wastewater, which is predicted to be at least 183–350 and expected to fluctuate drastically over time. The true species richness is likely even higher as the isolation, DNA extraction, library construction, and genome assembly method as well as the choice of a host all are liable to reduce the number of phages detected. Ninety-two of the newly isolated coliphages represent novel species of seven families; *Myoviridae, Siphoviridae, Podoviridae, Drexlerviridae, Chaseviridae,* and *Microviridae*. Though most of them distribute into 18 established genera, the diversity of these many phages isolated from a single strain is notable. They vary greatly in genome size and have a broad GC content range.

Apart from the analyses applied, the main difference between this and the comprehensive Korf et al., study from 2019 [23] is the isolation approach. Korf et al., isolated 50 phages from various sample types over several years from a wide collection of clinical *E. coli* isolates, whereas the wastewater sample collection and phage isolation in this study were performed in a matter of weeks on a single strain of *E. coli*. Still, the distribution of phage types including many of the same genera and the discovery of a handful of phages with limited similarity to known phages are in many aspects comparable, suggesting that the method of isolation (plaque purification) is perhaps the key limiting factor for uncovering the diversity of coliphages. However, fewer than 60% of the 104 coliphages are represented in the assessed metaviromes, emphasising the importance of cultivating phages to uncover the true diversity.

These findings add to our understanding of phage ecology and diversity, and through classification of these many phages we come yet another step closer to a more refined taxonomic understanding of phages. Furthermore, the numerous and diverse phages isolated in this study, all lytic to the same single strain, serve as an excellent opportunity to learn important phage-host interactions in future studies. These include, but are not limited to, lysogen-induced phage immunity, host-range, and anti-RE systems.

Finally, the first genus representative for at least three novel genera was sequenced in this study. *Skarprettervirus* and *Sortsnevirus* of the *Podoviridae* have been accepted by the ICTV. We propose that Halfdan is the type species of a new *Siphoviridae* genus, that the four novel *Myoviridae* species muut, alia, outra, and inny together with five unclassified *Myoviridae* also represent a new genus, and as the *Drexlerviridae* Jahat cannot with confidence be assigned to any described genera, Jahat may also represent its own lineage. In conclusion, this study shows that uncharted territory remains for even well-studied phage hosts and that cultivation approaches uncover vital genomes that seem absent from metagenomic studies.

## Figures and Tables

**Table 1:** List of 104 unique *Escherichia* phage species identified in 94 Danish wastewater samples. Phages (*n*) denotes the number of phages isolated with more than 95% nucleotide sequence similarity. Similarity is sequence identity (%) times sequence coverage (%) to closest relative (Blastn). Taxonomy is based on similarity (BLASTn) to closest related.

Phage	Accession	Phages (*n*)	Genome (bp)	ORFs	tRNAs	GC (%)	Family; Genus	Similarity (%)	Closest Relative	Accession
tootiki	MN850647	1	88,257	128	22	39	*Myoviridae; Felixounavirus*	90.2	Escherichia phage vB_EcoM_Alf5	NC_031082.1
mio	MN850631	1	83,431	121	18	39.1	*Myoviridae; Felixounavirus*	89.7	Salmonella virus VSe11	MG251391.1
allfine	MN850633	1	86,963	125	20	39	*Myoviridae; Felixounavirus*	91.2	Escherichia phage vB_EcoM-AYO145A	NC_028825.1
bumzen	MN850635	3	87,360	126	20	39.1	*Myoviridae; Felixounavirus*	92.5	Escherichia phage vB_EcoM_Alf5	NC_031082.1
dune	MN850636	1	88,511	129	20	39	*Myoviridae; Felixounavirus*	91.5	Escherichia phage vB_EcoM_VpaE1	NC_027337.1
warpig	MN850637	1	86,106	127	17	39	*Myoviridae; Felixounavirus*	93	Escherichia phage vB_EcoM_VpaE1	NC_027337.1
radambza	MN850639	1	86,702	127	19	38.9	*Myoviridae; Felixounavirus*	91.6	Escherichia phage vB_EcoM_VpaE1	NC_027337.1
ekra	MN850644	1	87,282	128	20	38.9	*Myoviridae; Felixounavirus*	92.9	Escherichia phage vB_EcoM_Alf5	NC_031082.1
humlepung	MN850564	3	85,311	119	19	39.1	*Myoviridae; Felixounavirus*	92.1	Escherichia phage vB_EcoM_VpaE1	NC_027337.1
finno	MN850619	1	87,554	129	20	38.9	*Myoviridae; Felixounavirus*	89.7	Escherichia phage vB_EcoM-AYO145A	NC_028825.1
garuso	MN850566	2	85,798	130	20	38.9	*Myoviridae; Felixounavirus*	90.9	Escherichia phage vB_EcoM-AYO145A	NC_028825.1
momo	MN850580	1	88,168	130	20	39	*Myoviridae; Felixounavirus*	90.7	Escherichia phage vB_EcoM-AYO145A	NC_028825.1
heid	MN850577	**6**	87,590	126	20	39	*Myoviridae; Felixounavirus*	91.2	Escherichia phage vB_EcoM_Alf5	NC_031082.1
skuden	MN850585	1	87,263	131	20	38.9	*Myoviridae; Felixounavirus*	91.1	Escherichia phage vB_EcoM_VpaE1	NC_027337.1
pinkbiff	MN850603	1	88,814	129	20	39	*Myoviridae; Felixounavirus*	93.9	Escherichia phage vB_EcoM_Alf5	NC_031082.1
fjerdesal	MN850605	3	87,715	128	21	39	*Myoviridae; Felixounavirus*	90.6	Escherichia phage vB_EcoM_AYO145A	NC_028825.1
andreotti	MN850610	1	83,391	117	20	39.2	*Myoviridae; Felixounavirus*	91.9	Escherichia phage vB_EcoM_VpaE1	NC_027337.1
nataliec	MN850611	2	89,137	134	20	39	*Myoviridae; Felixounavirus*	90.3	Escherichia phage vB_EcoM_AYO145A	NC_028825.1
adrianh	MN850614	2	88,226	128	19	38.9	*Myoviridae; Felixounavirus*	91.1	Escherichia phage vB_EcoM_Alf5	NC_031082.1
mistaenkt	MN850587	1	86,664	128	22	47.2	*Myoviridae; Suspvirus*	91.1	Escherichia phage SUSP2	NC_028935.2
nimi	MN850626	1	137,039	213	5	43.7	*Myoviridae; Vequintavirus*	93.3	Escherichia phage LL12	MH491969.1
navn	MN850642	1	141,707	224	4	43.6	*Myoviridae; Vequintavirus*	91.1	Escherichia coli O157 typing phage 4	KP869102.1
nomine	MN850649	1	137,991	220	5	43.6	*Myoviridae; Vequintavirus*	91.5	Escherichia phage LL12	MH491969.1
naswa	MN850595	1	138,583	222	5	43.6	*Myoviridae; Vequintavirus*	93.1	Escherichia phage LL12	MH491969.1
naam	MN850630	1	137,129	215	5	43.7	*Myoviridae; Vequintavirus*	94.5	Escherichia coli O157 typing phage 4	KP869102.1
ime	MN850576	2	137,114	217	5	43.6	*Myoviridae; Vequintavirus*	93.1	Escherichia phage LL12	MH491969.1
magaca	MN850612	1	135,826	217	5	43.6	*Myoviridae; Vequintavirus*	96	Escherichia phage slur12	LN881735.1
nom	MN850646	1	136,114	213	5	43.6	*Myoviridae; Vequintavirus*	92.6	Escherichia phage LL12	MH491969.1
isim	MN850597	1	138,289	219	5	43.6	*Myoviridae; Vequintavirus*	93.8	Escherichia phage LL12	MH491969.1
nomo	MN850578	1	137,702	218	5	43.7	*Myoviridae; Vequintavirus*	93.3	Escherichia phage APCEc02	NC_041869.1
inoa	MN850593	1	138,710	220	5	43,6	*Myoviridae; Vequintavirus*	92	Escherichia phage APCEc02	NC_041869.1
pangalan	MN850627	3	136,944	215	5	43.7	*Myoviridae; Vequintavirus*	94.8	Escherichia phage vB_EcoM_FFH2	NC_024134.1
tuntematon	MN850618	2	150,473	279	11	39.1	*Myoviridae; Phapecoctavirus*	89.6	Escherichia phage phAPEC8	NC_020079.1
anhysbys	MN850648	1	149,335	271	11	39.1	*Myoviridae; Phapecoctavirus*	91.5	Escherichia phage phAPEC8	NC_020079.1
ukendt	MN850565	1	150,947	266	11	39	*Myoviridae; Phapecoctavirus*	88.7	Escherichia phage phAPEC8	NC_020079.1
nepoznato	MN850571	4	151,514	265	10	38.9	*Myoviridae; Phapecoctavirus*	85.6	Escherichia phage phAPEC8	NC_020079.1
nieznany	MN850598	1	144,998	254	11	39.1	*Myoviridae; Phapecoctavirus*	88.9	Escherichia phage phAPEC8	NC_020079.1
muut	MN850573	1	146,307	243	13	37.4	*Myoviridae*	92	Escherichia phage vB_EcoM_PHB05	MF805809.1
alia	MN850632	1	147,009	246	13	37.5	*Myoviridae*	93.1	Enterobacteria phage ECGD1	KU522583.1
outra	MN850645	1	145,482	246	13	37.4	*Myoviridae*	93.8	Enterobacteria phage ECGD1	KU522583.1
inny	MN850601	1	147,483	247	13	37.4	*Myoviridae*	92.4	Enterobacteria phage ECGD1	KU522583.1
arall	MN850584	1	145,715	242	13	37.4	*Myoviridae*	94.6	Escherichia phage vB_vPM_PD06	MH816848.1
kvi	MN850615	1	163,673	266	-	40.5	*Myoviridae; Krischvirus*	94.2	Escherichia phage ECD7	NC_041936.1
kaaroe	MN850574	1	163,719	267	-	40.5	*Myoviridae; Krischvirus*	94.7	Enterobacteria phage RB49	NC_005066.1
dhabil	MN850621	1	165,644	266	3	39.5	*Myoviridae; Dhakavirus*	87.5	Enterobacteria phage JS10	NC_012741.1
dhaeg	MN850609	2	170,817	278	3	39.4	*Myoviridae; Dhakavirus*	87.4	Enterobacteria phage JS10	NC_012741.1
mogra	MN850579	1	168,724	263	2	37.7	*Myoviridae; Mosigvirus*	91.1	Escherichia phage vB_EcoM_PhAPEC2	NC_024794.1
mobillu	MN850622	1	163,063	255	2	37.7	*Myoviridae; Mosigvirus*	94.5	Escherichia phage p000y	MK047718.1
moha	MN850590	1	168,676	267	2	37.6	*Myoviridae; Mosigvirus*	94.8	Escherichia phage APCEc01	NC_029091.1
moskry	MN850651	1	169,410	269	2	37.6	*Myoviridae; Mosigvirus*	93.2	Escherichia virus vB_Eco_mar005P1	LR027383.1
teqskov	MN895437	1	165,017	257	6	35.4	*Myoviridae; Tequatrovirus*	91.7	Yersinia phage phiD1	NC_027353.1
teqdroes	MN895438	1	166,833	269	10	35.4	*Myoviridae; Tequatrovirus*	88.6	Escherichia phage T2	LC348380.1
teqhad	MN895434	1	167,892	270	10	35.3	*Myoviridae; Tequatrovirus*	90.1	Escherichia phage T2	LC348380.1
teqhal	MN895435	2	168,070	266	11	35.4	*Myoviridae; Tequatrovirus*	93.9	Escherichia phage slur13	LN881737.1
teqsoen	MN895436	1	166,468	268	10	35.5	*Myoviridae; Tequatrovirus*	91.7	Yersinia phage phiD1	NC_027353.1
flopper	MN850594	1	52,092	78	1	44.2	*Chaseviridae; Carltongylesvirus*	87	Escherichia phage ST32	NC_047830.1
damhaus	MN850602	1	51,154	89	-	44.1	*Drexlerviridae; Hanrivervirus*	85.8	Shigella phage pSf-1	NC_021331.1
herni	MN850640	2	50,971	89	-	44.1	*Drexlerviridae; Hanrivervirus*	87.6	Shigella phage pSf-1	NC_021331.1
grams	MN850567	1	49,530	83	-	44.1	*Drexlerviridae; Hanrivervirus*	87.1	Shigella phage pSf-1	NC_021331.1
aaroes	MN850572	1	51,662	92	-	44.1	*Drexlerviridae; Hanrivervirus*	83	Shigella phage pSf-1	NC_021331.1
aalborv	MN850591	1	46,660	79	-	43.9	*Drexlerviridae; Hanrivervirus*	86.9	Shigella phage pSf-1	NC_021331.1
haarsle	MN850600	2	48,613	85	-	44	*Drexlerviridae; Hanrivervirus*	87.1	Shigella phage pSf-1	NC_021331.1
egaa	MN850607	1	51,643	87	-	44.1	*Drexlerviridae; Hanrivervirus*	89.7	Shigella phage pSf-1	NC_021331.1
vojen	MN850569	1	50,709	86	-	44.1	*Drexlerviridae; Hanrivervirus*	89.7	Shigella phage pSf-1	NC_021331.1
tiwna	MN850643	1	51,014	85	-	44.6	*Drexlerviridae; Warwickvirus*	87.2	Escherichia phage vB_Eco_Swan01	NC_048202.1
tonijn	MN850641	2	51,627	86	-	44.6	*Drexlerviridae; Warwickvirus*	88.4	Escherichia phage vB_Eco_Swan01	NC_048202.1
tonnikala	MN850613	1	51,277	86	-	44.8	*Drexlerviridae; Warwickvirus*	86.4	Escherichia phage vB_Eco_Swan01	NC_048202.1
atuna	MN850620	1	50,732	88	-	44.6	*Drexlerviridae; Warwickvirus*	84.9	Escherichia virus vB_Eco_mar001J1	NC_048204
tunus	MN850638	1	51,111	87	-	44.8	*Drexlerviridae; Warwickvirus*	93.7	Escherichia phage SECphi27	NC_047938.1
orkinos	MN850586	2	49,798	81	-	44.6	*Drexlerviridae; Warwickvirus*	91.3	Escherichia phage SECphi27	NC_047938.1
ityhuna	MN850582	1	50,768	86	-	44.7	*Drexlerviridae; Warwickvirus*	93.3	Escherichia phage SECphi27	NC_047938.1
tonn	MN850596	2	51,012	87	-	44.5	*Drexlerviridae; Warwickvirus*	94	Escherichia phage vB_Eco_Swan01	NC_048202.1
tinuso	MN850634	1	50,856	86	-	44.8	*Drexlerviridae; Warwickvirus*	97.3	Escherichia phage vB_Eco_Swan01	NC_048202.1
tunzivis	MN850604	1	50,596	84	-	44.6	*Drexlerviridae; Warwickvirus*	94.5	Escherichia phage SECphi27	NC_047938.1
tuinn	MN850606	1	50,505	86	-	44.7	*Drexlerviridae; Warwickvirus*	94.8	Escherichia phage vB_Eco_Swan01	NC_048202.1
Jahat	MK552105	1	51,101	87	-	45.7	*Drexlerviridae*	68.5	Escherichia phage vB_Eco_Swan01	NC_048202.1
bob	MN850628	1	45,252	63	-	54.5	*Siphoviridae; Dhillonvirus*	88.6	Escherichia phage SECphi18	LT960609.1
mckay	MN850629	1	44,443	63	-	54.5	*Siphoviridae; Dhillonvirus*	83.8	Escherichia phage slur05	NC_028901.1
jat	MN850650	1	44,417	63	-	54.5	*Siphoviridae; Dhillonvirus*	89.4	Escherichia phage Gluttony	NC_031113.1
rolling	MN850575	1	46,017	64	-	54.2	*Siphoviridae; Dhillonvirus*	80.2	Escherichia phage Sloth	KX534339.1
welsh	MN850589	2	45,207	62	-	54.6	*Siphoviridae; Dhillonvirus*	83.8	Escherichia phage B2	KX534339.1
buks	MN850616	1	40,308	62	-	49.7	*Siphoviridae; Jerseyvirus*	91.3	Salmonella phage vB_SenS-Ent1	NC_019539.1
Skure	MK672798	1	59,474	92	-	44.6	*Siphoviridae; Seuratvirus*	90.4	Escherichia phage vB_Eco_SLUR25	LT907986.1
Halfdan	MH362766	1	42,858	57	-	53.7	*Siphoviridae*	28.8	Pseudomonas phage vB_PaeS_SCUT-S3	MK165657.1
Lilleen	MK629526	1	5342	6	-	46.9	*Microviridae; Gequatrovirus*	93.8	Escherichia phage SECphi17	LT960607.1
Lilleput	MK629525	1	5490	6	-	47	*Microviridae; Gequatrovirus*	93.4	Escherichia phage SECphi17	LT960607.1
Lilleto	MK629529	3	5492	6	-	46.8	*Microviridae; Gequatrovirus*	92.7	Escherichia phage SECphi17	LT960607.1
Lilledu	MK791318	1	5483	6	-	47.2	*Microviridae; Gequatrovirus*	92.6	Escherichia phage SECphi17	LT960607.1
lillemer	MN850599	1	5492	6	-	47.1	*Microviridae; Gequatrovirus*	94.5	Escherichia phage SECphi17	LT960607.1
Lilleven	MK629527	1	6090	9	-	44.4	*Microviridae; Alphatrevirus*	93.9	Enterobacteria phage St-1	NC_012868.1
sortsyn	MN850623	1	42,116	61	-	59	*Podoviridae; Murrayvirus*	92.3	Enterobacteria phage IME_EC2	KF591601.1
sortregn	MN850588	1	38,200	53	-	59.3	*Podoviridae; Murrayvirus*	97.3	Salmonella phage Lumpael	NC_048113.1
Skarpretter	MK105855	1	42,042	63	-	55.8	*Podoviridae; Skarprettervirus*	37.9	Escherichia phage C130_2	MH363708.1
sortkaff	MN850581	1	42,538	61	-	59.5	*Podoviridae; Sortsnevirus*	89.8	Klebsiella phage vB_KpnS_IME279	MF614100.1
Sortsne	MK651787	1	41,912	62	-	60	*Podoviridae; Sortsnevirus*	67.6	Klebsiella phage vB_KpnS_IME279	MF614100.1
aldrigsur	MN850592	1	42,379	55	-	55.7	*Autographviridae; Bonnellvirus*	71.9	Enterobacteria phage J8-65	NC_025445.1
altidsur	MN850568	1	42,197	53	-	55.7	*Autographviridae; Bonnellvirus*	71.8	Enterobacteria phage J8-65	NC_025445.1
forsur	MN850617	1	42,476	56	-	55.4	*Autographviridae; Bonnellvirus*	72	Enterobacteria phage J8-65	NC_025445.1
glasur	MN850583	1	42,507	56	-	55.4	*Autographviridae; Bonnellvirus*	72.3	Enterobacteria phage J8-65	NC_025445.1
Lidtsur	MK629528	1	42,291	56	-	54.6	*Autographviridae; Bonnellvirus*	69	Enterobacteria phage J8-65	NC_025445.1
megetsur	MN850608	1	42,132	54	-	55.8	*Autographviridae; Bonnellvirus*	73.1	Enterobacteria phage J8-65	NC_025445.1
mellemsur	MN850570	1	40,770	50	-	55.8	*Autographviridae; Bonnellvirus*	76.4	Enterobacteria phage J8-65	NC_025445.1
smaasur	MN850625	1	41,110	50	-	55.4	*Autographviridae; Bonnellvirus*	93.3	Enterobacteria phage J8-65	NC_025445.1
usur	MN850624	2	41,906	51	-	55.4	*Autographviridae; Bonnellvirus*	73.3	Enterobacteria phage J8-65	NC_025445.1

## Supplementary Materials

The following are available online at https://www.mdpi.com/1999-4915/12/9/986/s1, Figure S1: VIRIDIC Plot of intergenomic sequence similarity, aligned genome fraction and genome length ratio for the 104 coliphage species, Figure S2: Distribution of the 136 coliphage species per WWTP location (urban vs rural), Figure S3: Phylogenetic and genomic analyses of the six *Microviridae* coliphages, Figure S4: Phylogenomic nucleotide distances (Gegenees, BLASTn) for the *Hanrivervirus* coliphages, Figure S5: Phylogenomic nucleotide distances (Gegenees, BLASTn) for phage Halfdan, Figure S6: Phylogenetic and genomic analyses of the *Bonnellvirus* coliphages, Figure S7: Phylogenomic nucleotide distances (Gegenees, BLASTn) for the *Podoviridae* coliphages, Figure S8: Mapped reads from the Wang et al. (2019) study to the genomes of flopper and Lilleven, Figure S9: Alignment coverage distribution of IMG/VR iVGs to the 104 coliphage genomes and significant hits to these, Table S1: List of phages per wastewater sample and corresponding sequence result metrics, Table S2: AMG screening results, Table S3: Species richness estimations, Table S4: Read mapping results for samples with *Microviridae,* Table S5: List of sequence data assessed for matches with the 104 coliphage genomes, Table S6: Mapped reads from the Wang et al. (2019) study and the IMG/VR microbiomes, Table S7: IMG/VR and GVD blast results.
