# Supplementary material for: Exploring the Remarkable Diversity of Culturable Escherichia coli Phages in the Danish Wastewater Environment
Source: Viruses. 2020 Sep 4;12(9):986. doi: 10.3390/v12090986 (PMC7552041; doi:10.3390/v12090986)
Supplement: Supplementary file 1 [file viruses-12-00986-s001.zip › supplementary_NO.pdf]

Supplementary data:

Exploring the Remarkable Diversity of *Escherichia coli* Phages in the Danish Wastewater Environment, Including 92 Novel Phage Species

Figures:

S1 VIRIDIC Plot of intergenomic sequence similarity, aligned genome fraction and genome length ratio for the 104 coliphage species

S2 Distribution of the 136 coliphage species per WWTP location (urban vs rural)

S3 Phylogenetic and -genomic analyses of the six *Microviridae* coliphages

S4 Phylogenomic nucleotide distances (Gegenees, BLASTn) for the *Hanrivervirus* coliphages

S5 Phylogenomic nucleotide distances (Gegenees, BLASTn) for phage Halfdan

S6 Phylogenetic and -genomic analyses of the *Bonnellvirus* coliphages

S7 Phylogenomic nucleotide distances (Gegenees, BLASTn) for the *Podoviridae* coliphages

S8 Mapped reads from the Wang *et al.* (2019) study to the genomes of flopper and Lilleven

S9 Alignment coverage distribution of IMG/VR iVGs to the 104 coliphage genomes and significative hits to these

Tables:

S1 List of phages per wastewater sample and corresponding sequence metrics

S2 AMG screening results

S3 Species richness estimations

S4 Read mapping results for samples with *Microviridae*

S5 List of sequence data assessed for matches with the 104 coliphage genomes

S6 Mapped reads from the Wang *et al.* (2019) study and the IMG/VR microbiomes, separate file: Table_S6.xlsx

S7 IMG/VR and GVD blast results, separate file: Table_S7.xlsx

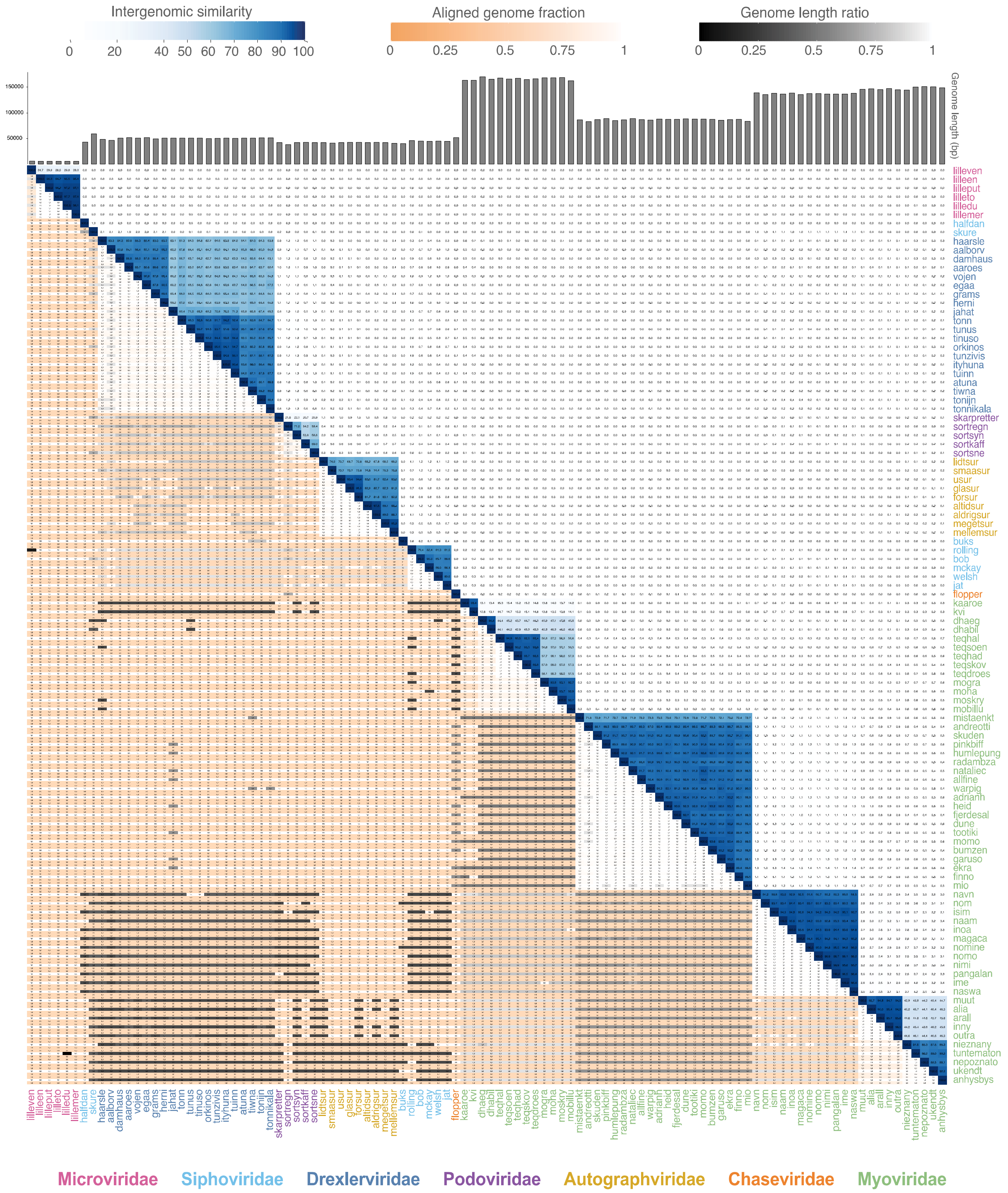

Figure S1 VIRIDIC plot of intergenomic sequence similarity, aligned genome fraction and genome length ration for the 104 coliphage species. Genome length is not calculated (NA) when the intergenomic similarity is zero.

A

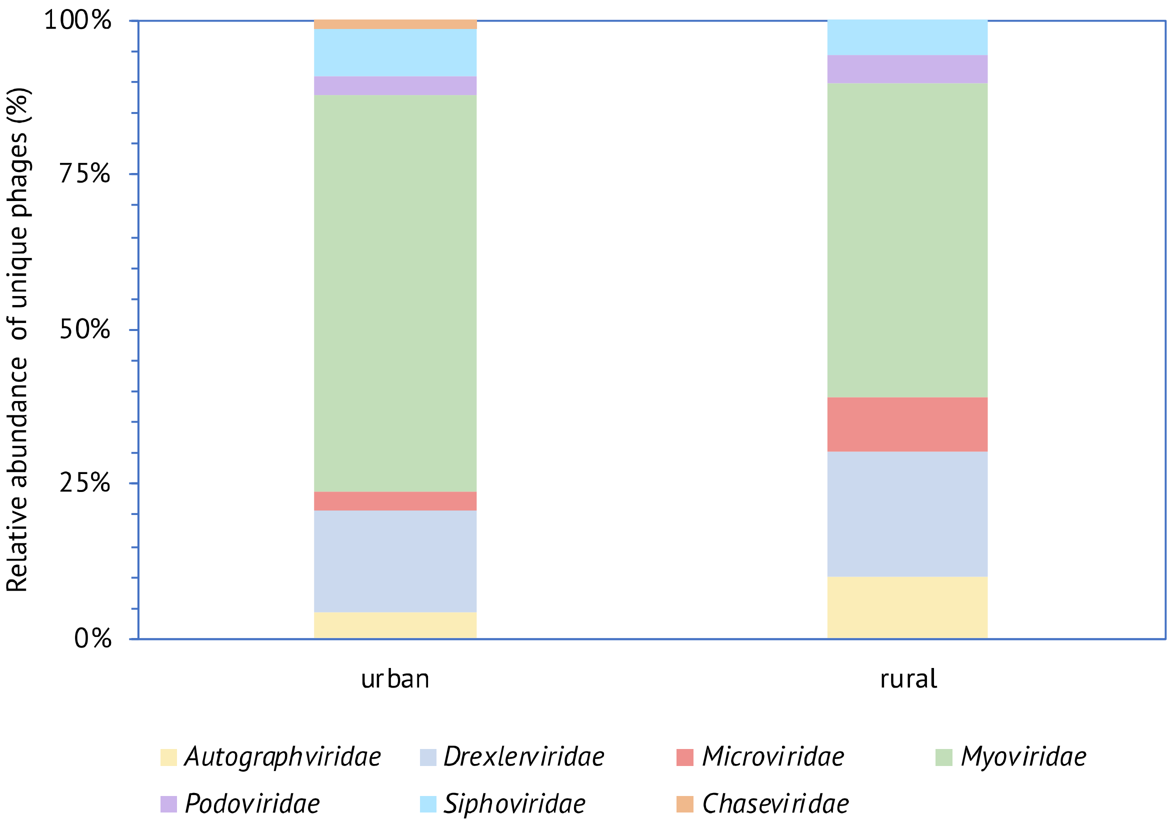

B

	Myoviridae	*Drexlerviridae*	*Autographviridae*	*Siphoviridae*	*Microviridae*	*Podoviridae*	*Chaseviridae*	total phages
urban	43	14	3	5	2	2	1	70
rural	35	11	7	4	6	3	0	66

Figure S2 Distribution of the 136 unique phage species grouped by family per wastewater treatment plant location; urban (n = 22) or rural (n = 22). **A** as relative abundance per location type. **B** as true counts.

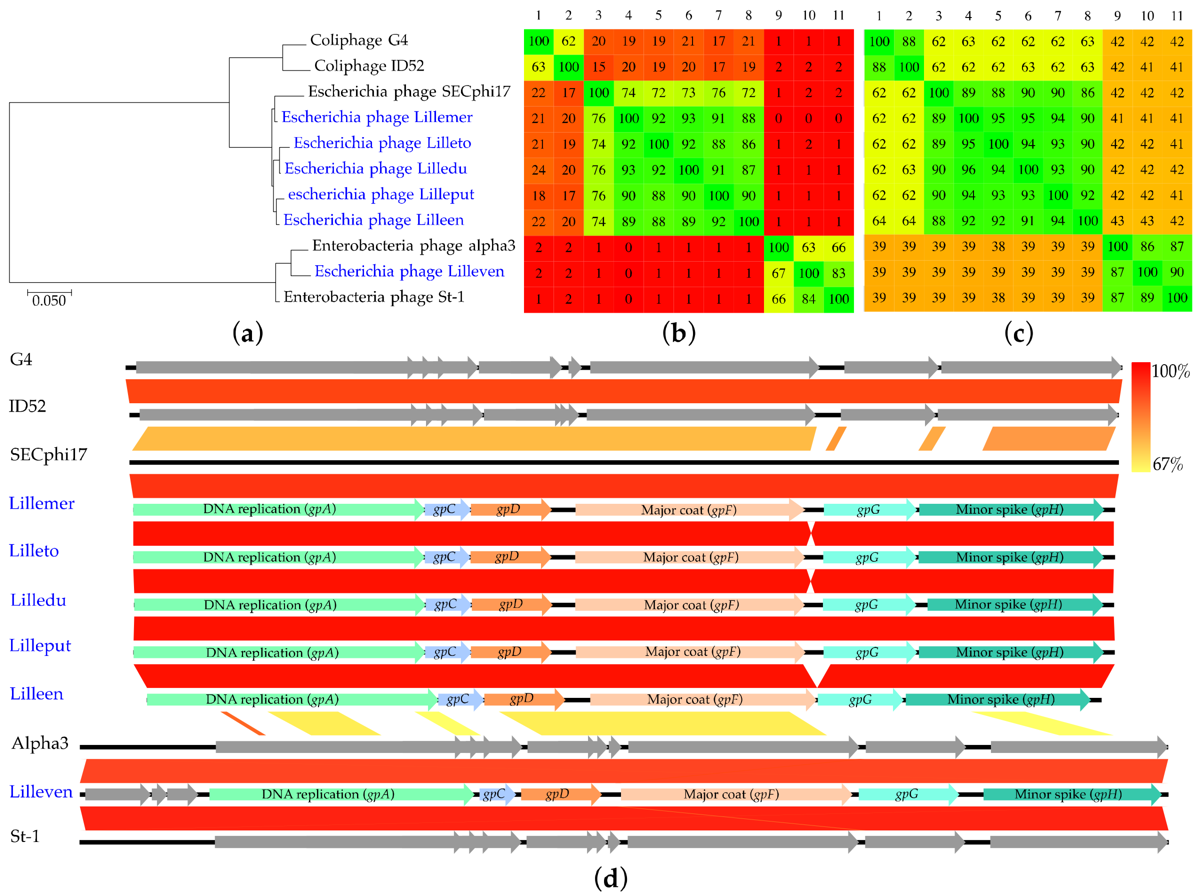

Figure S3 Phylogenetic and -genomic analyses of the *Microviridae* coliphages and closest relatives. **A** Phylogenetic tree (Maximum log likelihood: -6065.3, DNA replication protein gene *gpA*), scalebar: substitutions per site. **B** phylogenomic nucleotide distances (Gegenees, BLASTn: fragment sze: 200, step size: 100, threshold: 0%). **C** phylogenomic amino acid distances (Gegenees, BLASTx: fragment sze: 200, step size: 100, threshold: 0%). **D** Pairwise genomic alignments (Easyfig, BLASTn), colour bars between genomes indicate present nucleotide similarity, genomes have been modified to have similar starting points. Blue font denotes phages isolated in this study.

A

		1	2	3	4	5	6	7	8	9
1	Damhaus	100	74	76	76	83	69	73	76	73
2	Herni	74	100	75	78	74	74	70	84	77
3	Grams	79	77	100	75	80	71	71	76	73
4	Vojen	77	78	74	100	74	73	72	80	77
5	Aaroes	82	73	77	72	100	68	70	74	71
6	Aalborv	76	80	74	79	76	100	74	78	75
7	Haarsle	77	73	73	75	74	70	100	76	75
8	Egaa	75	83	73	79	74	70	72	100	78
9	pSf-1	72	76	70	77	71	68	71	78	100

B

1	2	3	4	5	6	7	8	9
100	84	85	85	88	79	81	85	83
84	100	86	86	85	82	80	90	85
87	88	100	86	88	81	82	87	84
85	86	84	100	84	81	82	89	85
88	84	85	83	100	79	80	84	81
85	88	85	87	86	100	83	86	84
85	84	83	84	84	80	100	86	84
85	89	84	87	84	79	82	100	85
82	84	81	84	81	77	80	85	100

Figure S4 Phylogenomic nucleotide distances for the *Hanrivervirus* coliphages and type species pSf-1. **A** (Gegenees, BLASTn: fragment sze: 200, step size: 100, threshold: 0%). **B** phylogenomic amino acid distances (Gegenees, BLASTx: fragment sze: 200, step size: 100, threshold: 0%).

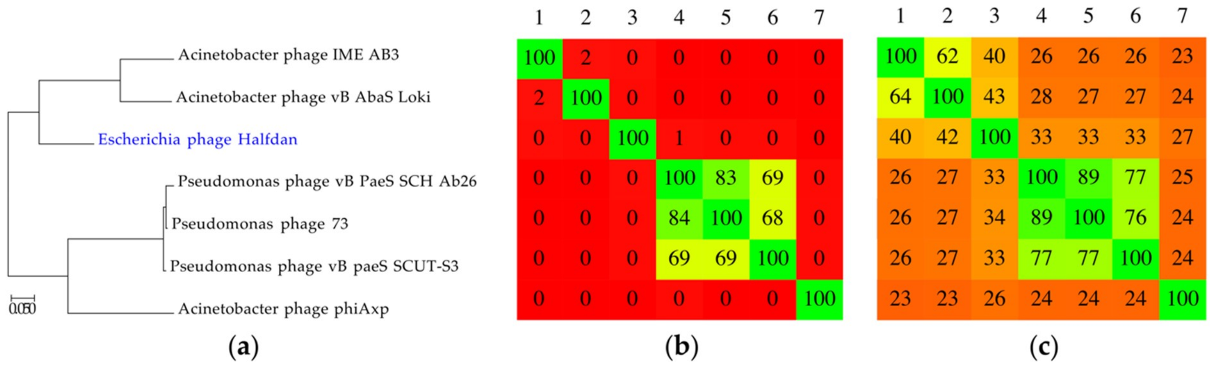

Figure S5 Phylogenetic and -genomic analyses of the *Siphoviridae* Halfdan and closest relatives. Blue font denotes phages isolated in this study. **A** Phylogenetic tree (Maximum log likelihood: -7678.71, large terminase subunit gene *TerL*), scalebar: substitutions per site. **B** phylogenomic nucleotide distances (Gegenees, BLASTn: fragment sze: 200, step size: 100, threshold: 0%). **C** phylogenomic amino acid distances (Gegenees, BLASTx: fragment sze: 200, step size: 100, threshold: 0%).

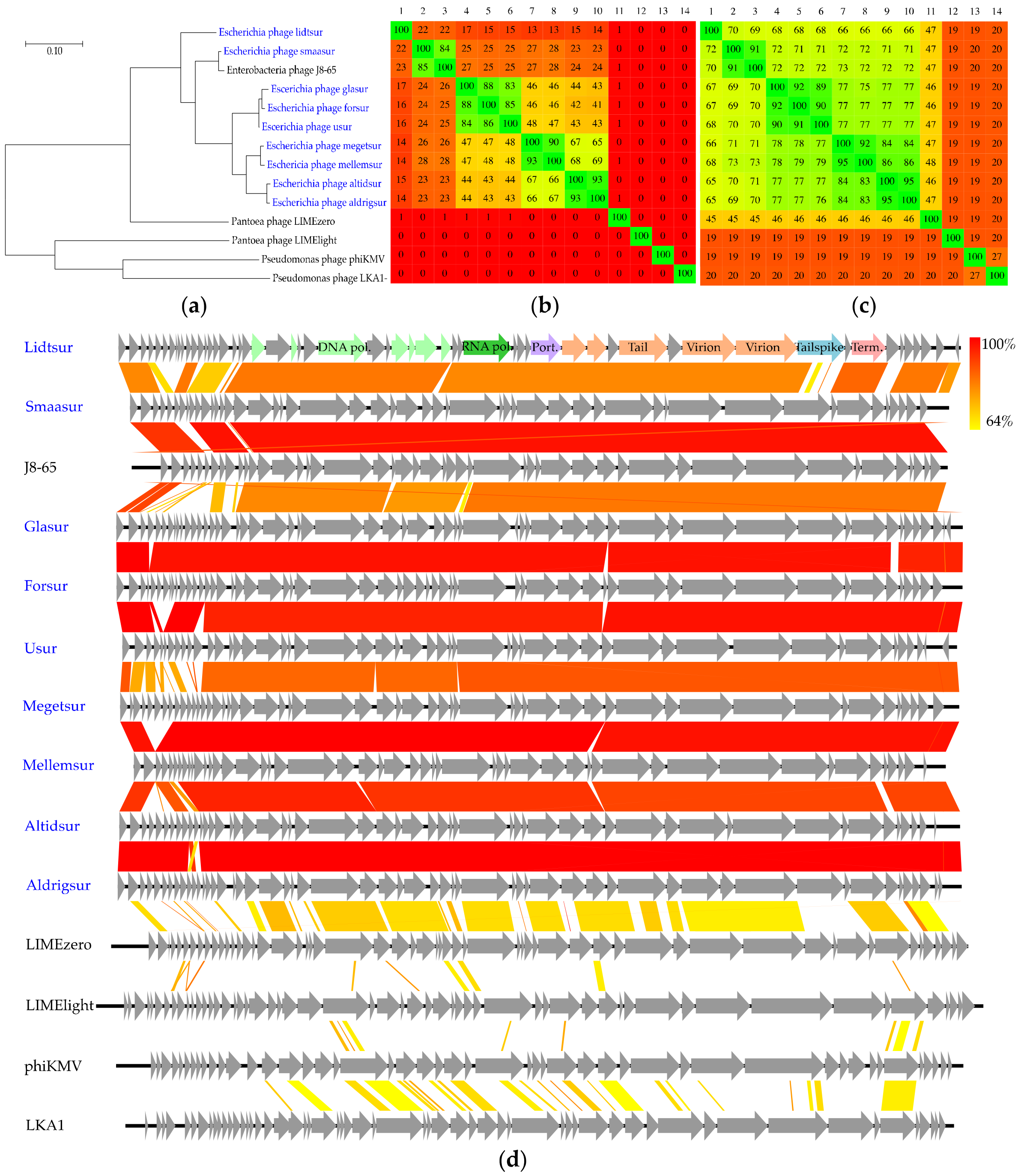

Figure S6 Phylogenetic and -genomic analyses of the *Bonnellvirus* coliphages and closest relatives. **A** Phylogenetic tree (Maximum log Likelihood: -11728.26, large terminase subunit), scalebar: substitutions per site. **B** Phylogenomic nucleotide distances (Gegenees, BLASTn: fragment size: 200, step size: 100, threshold: 0%). **C** Phylogenomic amino acid distances (Gegenees, tBLASTx: fragment size: 200, and step size: 100, threshold: 0%). **D** Pairwise alignment of phage genomes (Easyfig, BLASTn), the colour bars between genomes indicate percent pairwise similarity. Genomes have been modified to have similar starting points. Blue font denotes phages isolated in this study.

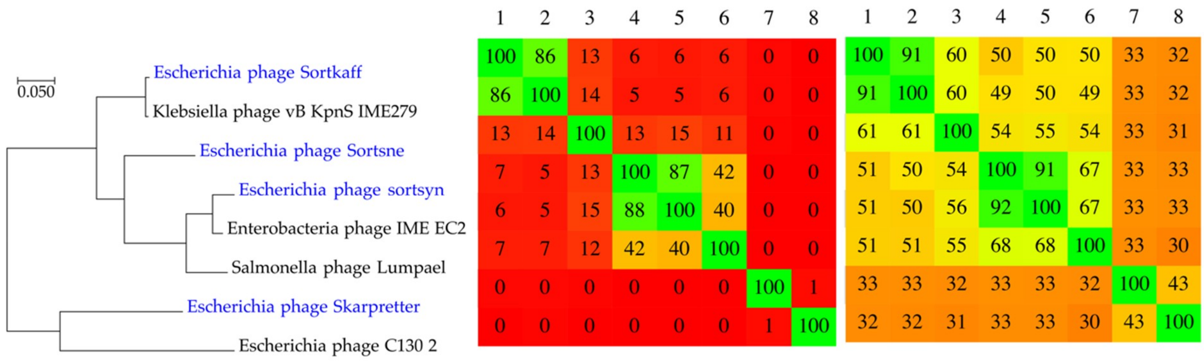

(**a**) (**b**) (**c**)

Figure S7 Phylogenetic and -genomic analyses of the *Podoviridae* coliphages and closest relatives. Blue font denotes phages isolated in this study. **A** Phylogenetic tree (Maximum log likelihood: -8023.43, large terminase subunit gene *TerL*), scalebar: substitutions per site. **B** phylogenomic nucleotide distances (Gegenees, BLASTn: fragment sze: 200, step size: 100, threshold: 0%). **C** phylogenomic amino acid distances (Gegenees, BLASTx: fragment sze: 200, step size: 100, threshold: 0%).

Escherichia phage flopper

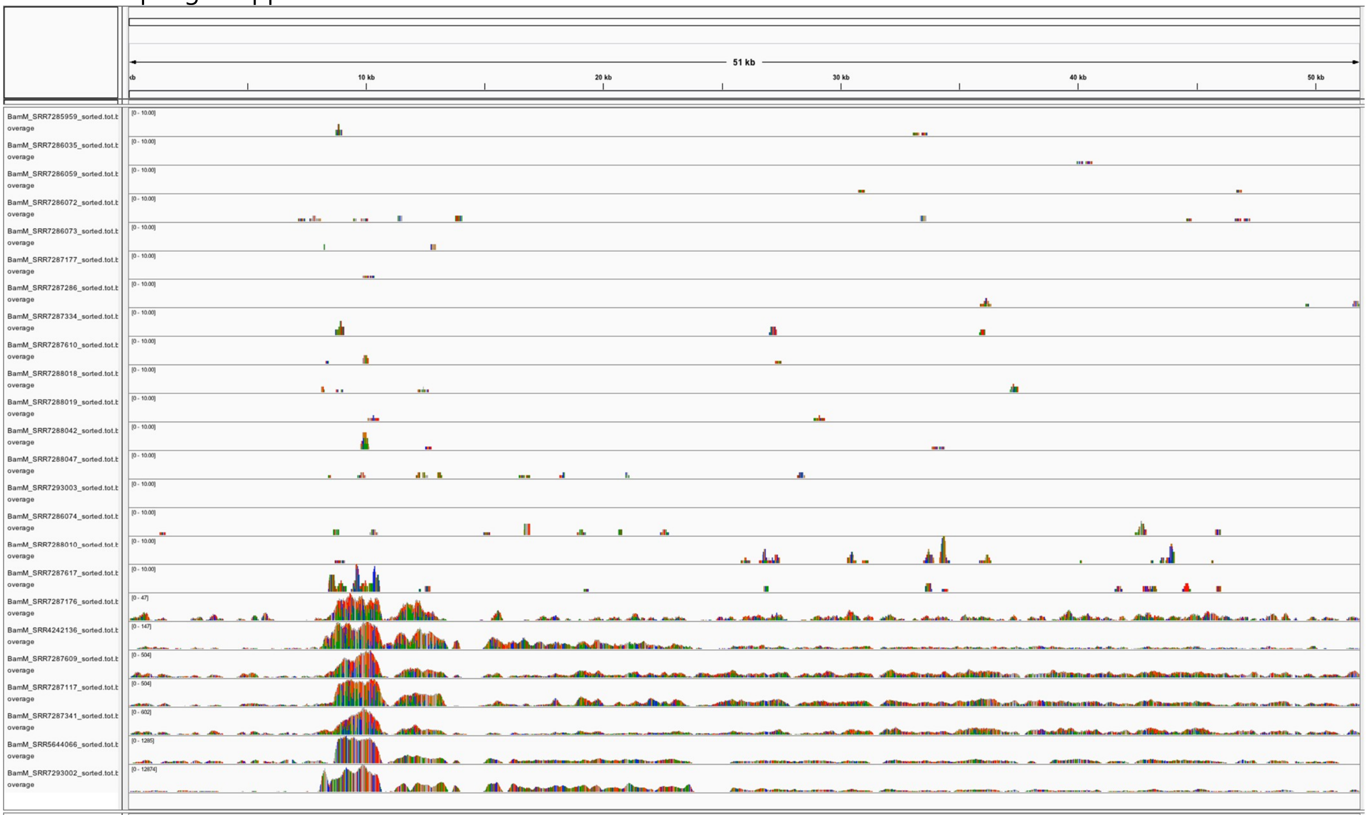

Escherichia phage lilleven

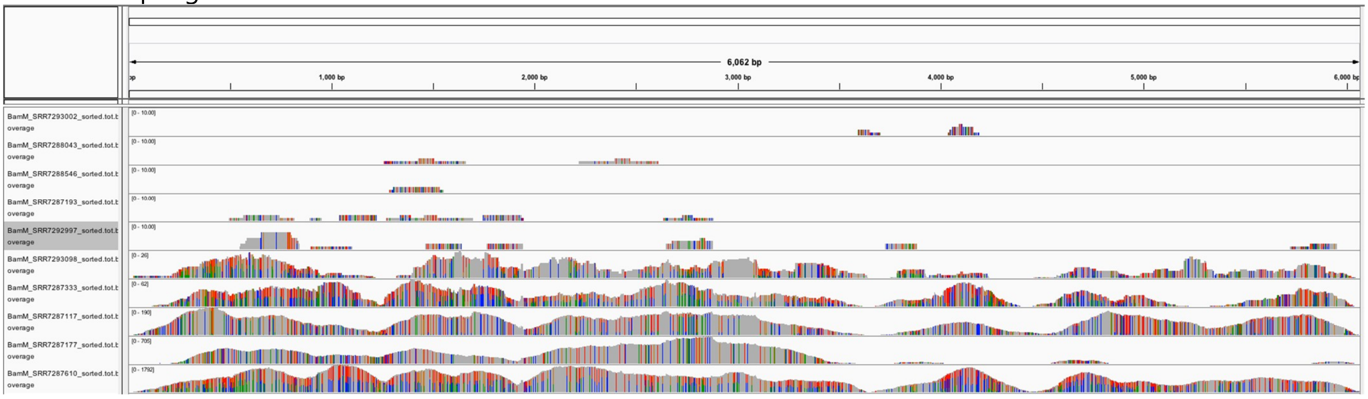

Figure S8 Mapped reads from the Wang *et al.* (2019) study to the genomes of flopper (top) and Lilleven (bottom). Flopper: The last seven rows correspond to the seven samples were close relatives of flopper were identified (>70% coverage), sample SRR728761 has a coverage of 0.08% and SRR7288010 of 0.08%. Lilleven: the last five rows correspond to the five samples were close relatives of Lilleven was identified (>70% coverage), sample SRR7292997 has a coverage of 0.24% and SRR728871 of 0.23%. In samples were close relatives of Lilleven was found the coverages has Gaussian distribution, in the others the mapping regions have blunt ends.

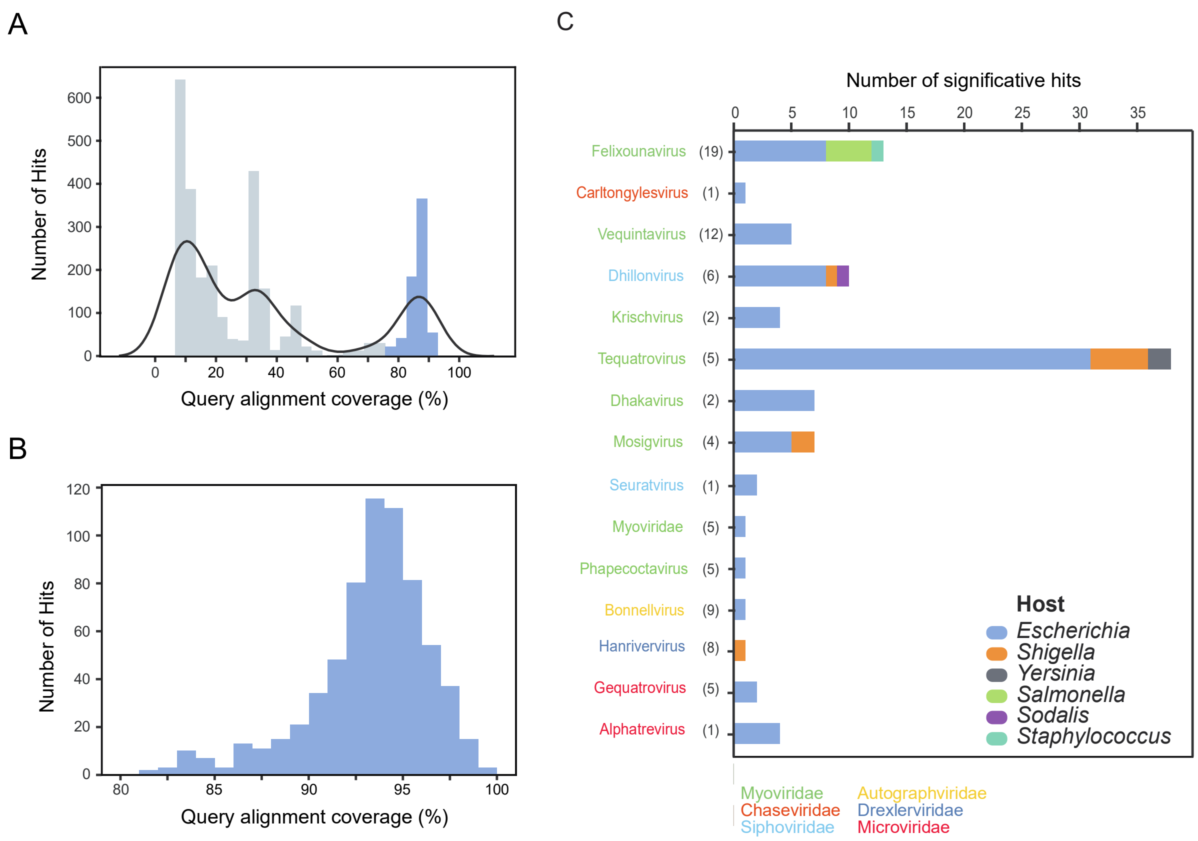

Figure 9 **A** Blast query sequence coverage distribution of BLASTn searches of coliphages against a database of the Integrated Microbial Genomes/Virus IMG/VR and Gut Virome Database (GVD) sequences. Significative hits per coliphage sequence covering ⩾80% of coliphage genomes are shown in blue. **B** A close-up of blast query coverage distribution hits ⩾80% **C** Significative hits (>80% query coverage) counts per coliphage taxonomic group against iVGs sequences from the IMG/VR databases. Each bar represents the matches to individual coliphage groups and are color-coded according to taxonomic family. The number of phages within each group is written within parentheses.

Table S1 List of positive samples (n = 96) and phages isolated from these with corresponding sequence metrics i.e. contigs, reads and assembled phage genome per sample (n = 104), as well as coverage per genome assembly.

Sample	Location	Contigs	Reads	Phages (n)	Phage[1]	Coverage (x)
1A	Billund	4	72371	1	buks	256
1B	Billund	187	407029	2	dhabil	253
					mobillu	46
2A	Grindsted	270	306434	1	pangalan	262
3A	Avedøre	1963	294281	1	fjerdesal-like	302
3D	Avedøre	4254	386918	1	tootiki	368
4B	Damhusåen	6157	481628	1	damhus	56
6A	Kolding	3986	168057	1	mistaenkt	112
7B	Esbjerg Vest	1721	508938	2	tuntematon	438
					atuna	52
7C	Esbjerg Vest	2	368607	1	tuntematon-like	345
8C	Esbjerg øst	4129	242842	2	heid-like	272
					tonn-like	22
10D	Skovlund	336	281474	1	teqskov	184
11B	Varde	6570	378556	2	sortsyn	213
					Lilleven	701
11C	Varde	1164	118310	2	nimi	51
					smaasur	28
12B	Drøsbro	4443	255665	1	humlepung-like	290
12C	Drøsbro	455	147560	2	bob	43
					ime-like	116
12D	Drøsbro	4691	361420	2	Lilleput	254
					teqdroes	192
13B	Hadsten	724	201166	1	mckay	385
13C	Hadsten	2466	257350	1	mio	398
13D	Hadsten	294	78995	1	alia	22
14A	Hammel	2495	140257	2	allfine	114
					tinuso	81
14B	Hammel	424	75810	1	bumzen-like	53
14D	Hammel			1	bumzem-like	22
15A	Hinnerup	4320	368108	2	bumzen	459
					Skarpretter	20
19C	Tørring	3833	264373	1	dune	220
19D	Tørring	37	105090	1	nepoznato-like	58
20A	Helsingør	744	143289	2	warpig	92
					tunus	29
20D	Helsingør	5180	2064920	1	radambza	1280
21B	Herning	5829	2405937	3	navn	141
					tiwna	438
					herni	196
21C	Herning	4208	772492	1	ekra	667
22A	Hillerød	224	210802	1	outra	127
22C	Hillerød	137	122673	1	anhysbys	97
23A	Lemvig	864	233926	2	welsh-like	64
					nomine	255
23C	Lemvig	5948	390078	2	jat	193
					Skure	226
24B	Bevtoft	4010	230626	1	heid-like	667
24C	Bevtoft	5548	595967	1	heid-like	667
25B	Gram	2314	950865	2	humlepung	998
					grams	121
25C	Gram	160	503468	2	mogra	310
					Lilledu	67
26A	Haderslev	3	203554	1	moha	99
26B	Haderslev	24	184597	1	teqhad	114
27A	Halk	2481	457114	2	teqhal-like	236

	Halk				usur-like	101
27D	Halk	1741	3069013	2	usur	378
27D	Halk				teqhal	1234
28D	Jegerup	36	55099	1	nom	30
29A	Nustrup	539	116036	1	garuso-like	53
29D	Nustrup	39	20948	1	rolling	31
30B	Over Jerstal	74	29055	2	orkinos	49
30B	Over Jerstal				Lidtsur	20
30D	Over Jerstal	5355	646801	2	herni-like	159
					orkinos-like	650
31B	Skovby	6327	2572603	2	isim	30
31B	Skovby				megetsur	89
31C	Skovby	5698	641691	1	finno	114
31D	Skovby	1334	198073	1	naam	44
32B	Skrydstrup	513	141211	1	tonijn-like	179
32C	Skrydstrup	1564	259226	2	tonijn	245
					moskry	63
32D	Skrydstrup			1	ukendt	438
33A	Sommersted	443	121856	2	garuso	114
					Lilleen	26
33D	Sommersted	343	88714	1	altidsur	50
34B	Vojens	2628	1542668	1	vojen	28
34D	Vojens	6421	4832796	3	heid-like	53
					Halfdan	12122
					mellemsur	3786
35A	Årøsund	3839	397068	2	nepoznato	120
					Jahat	428
35B	Årøsund	4312	319707	2	muut	158
					aaroes	165
35D	Årøsund	2896	268470	1	kaaroe	110
36B	Ejby Mølle	125	105836	1	ime	255
37A	Bogense	2633	240834	1	humlepung-like	998
37D	Bogense	4130	749600	1	heid	888
38B	Hofmansgav	3405	214442	1	heid-like	53
					nomo	102
38D	Hofmansgav	1160	263338	2	momo	114
39A	Hårselv	4968	428445	1	fjerdesal-like	404
39B	Hårselv	99	149737	2	sortkaff	52
					haarsle-like	100
39D	Hårselv	1557	195211	1	ityhuna	22
40A	Odense NV	214	64473	1	Sortsne	75
40D	Odense NV	340	102911	1	glasur	20
41B	Odense NØ	3033	1057676	1	arall	48
41D	Odense NØ	1708	193172	1	skuden	998
42C	Otterup	847	167020	1	adrainh-like	53
43B	Søndersø	157	157168	1	teqsoen	70
43C	Søndersø	153	34251	1	Lilleto	56
43D	Søndersø	338	146898	3	lilleto-like	226
					sortregn	145
					welsh	213
44B	Ålborg V	2368	1352388	3	lilleto-like	1006
					aalborv	877
					aldrigsur	525
44C	Ålborg V	2293	1992256	1	inoa	52
44D	Ålborg V	4873	1197754	1	flopper	86
45A	Ålborg Ø	4454	985167	1	nazwa	255
45C	Ålborg Ø	1215	1870976	4	haarsle	244
					tonn	354
					nieznany	166
					lillemer	1066
45D	Ålborg Ø	4109	1842928	2	inny	717

					pangalan-like	255
46A	Marselisborg	6441	91540	1	pinkbiff	686
46B	Marselisborg	6440	1073152	1	tunzivis	959
46C	Marselisborg	5689	737432	1	fjerdesal	404
46D	Marselisborg	5655	794015	1	tuinn	83
47A	Egå	2532	846912	2	egaa	210
					dhaeg	336
47B	Egå	7018	938687	1	andreotti	998
47C	Egå	1531	801644	1	dhaeg-like	311
47D	Egå	5278	717643	1	nataliec	404
48A	Viby	4859	1290861	3	nepoznato-like	153
					tonnikala	1556
					magaca	57
48C	Viby	3106	1292261	2	nataliec-like	404
					kvi	59
48D	Viby	5957	1063300	3	pangalan-like	255
					forsur	155
					nepoznato-like	64
48B	Viby	3194	916618	1	adrainh	686

[1]Phage genomes with >95% nucleotide similarity to other phage genomes in the dataset are named after these, e.g. the felixounavirus identified in sample 42C has >95% nucleotide sequence similarity with the felixounavirus adrianh and is consequently named adrainh-like. The "-like" phage genomes are so far not deposited in public databases.

Table S2 list of Auxiliary Metabolism Genes (AMGs) identified in the 104 unique wastewater Escherichia phages by a VIBRANT search. Including predicted function, KO numbers and counts.

Gene	Protein	KO number	Function in bacteriophages	Count	phages with this gene
rmlC	dTDP-4-dehydrorhamnose 3,5-epimerase	K01790	dtdp rhamnose biosynthesis	10	*Cluster VI and VII*
rmlA	glucose-1-phosphate thymidylyltransferase	K00973	dtdp rhamnose biosynthesis	8	*Cluster VII*, ukendt and tuntematon
rmlB	dTDP-glucose 4,6-dehydratase	K01710	dtdp rhamnose biosynthesis	8	*Cluster VII*, ukendt and tuntematon
rmlD	dTDP-4-dehydrorhamnose reductase	K00067	dtdp rhamnose biosynthesis	10	*Cluster VI and VII*
dcm	DNA (cytosine-5)-methyltransferase	K17398	DNA modification	5	*Cluster VII*
NAMPT	nicotinamide phosphoribosyltransferase	K03462	unknown	25	*Felixounavirus*, *Suspvirus* and *Cluster VII*
mec	CysO sulfur-carrier protein]-S-L-cysteine hydrolase	K21140	tail fiber protein	20	*Hanrivervirus* and unclassifed *Tunavirinae*
folA	dihydrofolate reductase	K00287	de novo synthesis of pyrimidines, thymidylic acid, and certain amino acids	33	*Felixounavirus, Suspvirus, Krischvirus, Dhakavirus, Mosigvirus* and *Tequatrovirus*
aroAG	3-deoxy-7-phosphoheptulonate synthase	K01626	unknown	3	Three of the four *Mosigvirus*
AUP1	UDP-N-acetylglucosamine diphosphorylase	K23144	DNA modification	4	*Mosigvirus*
KdsD	arabinose-5-phosphate isomerase	K06041	DNA modification	4	*Mosigvirus*
dcm	DNA (cytosine-5)-methyltransferase	K00558	DNA modification	1	Pangalan
queC	7-cyano-7-deazaguanine synthase	K06920	DNA modification	1	Skure
queE	7-carboxy-7-deazaguanine synthase	K10026	DNA modification	1	Skure
folE	GTP cyclohydrolase	K01495	DNA modification	1	Skure
queD	6-carboxy-5,6,7,8-tetrahydropterin synthase	K01737	DNA modification	1	Skure

Table S3 Rarefaction and extrapolation of the coliphage (n=136) dataset using iNEXT in RStudio. Code: m <- c(1, 100, 250, 500, 1000), iNEXT(dataset, datatype = "incidence_raw", size =m).

```
$iNextEst: diversity estimates with rarefied and extrapolated samples.
     t       method order      qD  qD.LCL  qD.UCL    SC SC.LCL SC.UCL
1    1 interpolated     0   1.483   1.233   1.733 0.008  0.004  0.013
2   88 interpolated     0 100.101  86.238 113.965 0.394  0.298  0.490
4   90 extrapolated     0 101.895  87.790 116.000 0.399  0.302  0.496
6  250 extrapolated     0 205.565 168.282 242.849 0.696  0.554  0.837
8 1000 extrapolated     0 307.553 190.873 424.233 0.987  0.957  1.000

$AsyEst: asymptotic diversity estimates along with related statistics.
                   Observed Estimator Est_s.e. 95% Lower 95% Upper
Species Richness    101.000   311.936   73.498   209.647   510.530
Shannon diversity    90.192   266.865   42.640   183.293   350.437
Simpson diversity    76.421   178.112   34.531   110.432   245.793
```

Table S4 List of reads in samples with *Microviridae* phages and percentage of reads mapping to *Microviridae* phage genomes and the E. coli K-12 MG1655 host reference genome NC_000913.

Sample	Phages per sample (n)	Microviridae phage	Total reads	Reads mapping to Microviridae (n)	Reads mapping to Microviridae (%)	Reads mapping to MG1655 (n)	Reads mapping to MG1655 (%)
11B	2	Lilleven	417724	35613	8.53	163123	39.05
12D	2	Lilleput	436506	10558	2.42	131734	30.18
25C	2	Lilledu	571338	3048	0.53	15801	2.77
33A	1	Lilleen	34918	1058	3.03	3336	9.55
43C	1	Lilleto	34251	2361	6.66	3685	10.39
45C	4	lillemer	2159874	50988	2.36	721646	33.41

Table S5 List sequence data assessed for matches with the 104 coliphage genomes.

Source	(n)	Reference
The Global Sewage Project: Inlet wastewater metagenomes	235	Hendriksen RS, Munk P, Njage P, et al (2019) Global monitoring of antimicrobial resistance based on metagenomics analyses of urban sewage. Nat Commun 10:1–12. https://doi.org/10.1038/s41467-019-08853-3
Danish wastewater libraries from a Sewage surveillance study	64	BioProject: PRJEB13832
Danish pig-gut metagenomes	96	Xiao L, Estellé J, Kiilerich P, et al (2016) A reference gene catalogue of the pig gut microbiome. Nat Microbiol 1:1–6. https://doi.org/10.1038/nmicrobiol.2016.161
Human gut metagenomes from Danish subjects	115	Li J, Wang J, Jia H, et al (2014) An integrated catalog of reference genes in the human gut microbiome. Nat Biotechnol 32:834–841. https://doi.org/10.1038/nbt.2942
Sequencing runs from fecal samples of ten healthy adults from Cork, Ireland	235	Shkoporov AN, Clooney AG, Sutton TDS, et al (2019) The Human Gut Virome Is Highly Diverse, Stable, and Individual Specific. Cell Host Microbe 26:527-541.e5. https://doi.org/10.1016/j.chom.2019.09.009
Sequencing runs from a Chinese study of metaviromes from humans, mammals, birds and water	38	Wang H, Ling Y, Shan T, et al (2019) Gut virome of mammals and birds reveals high genetic diversity of the family Microviridae. Virus Evol 5:1–8. https://doi.org/10.1093/ve/vez013

IMG/VR database: Uncultured viral genomes (UVIGs)	735 106	Paez-Espino D, Chen IMA, Palaniappan K, et al (2017) IMG/VR: A database of cultured and uncultured DNA viruses and retroviruses. Nucleic Acids Res 45:D457–D465. https://doi.org/10.1093/nar/gkw1030
IMG/VR database: Isolated virus genomes (iVGs)	8392	Paez-Espino D, Chen IMA, Palaniappan K, et al (2017) IMG/VR: A database of cultured and uncultured DNA viruses and retroviruses. Nucleic Acids Res 45:D457–D465. https://doi.org/10.1093/nar/gkw1030

Table S6 Mapped reads from the Wang *et al.,* (2019) study and the IMG/VR database microbiomes. SEPARATE FILE

Table S7 Blast results for the 104 coliphage genomes against the IMG/VR and GVD databases. SEPARATE FILE
